# Integrative analysis of hub genes for recurrent pregnancy loss with antiphospholipid syndrome: integrated bioinformatics analysis, machine learning and experimental validation

**DOI:** 10.3389/fimmu.2026.1783244

**Published:** 2026-06-04

**Authors:** Huan Zeng, Chengming Ding, Weilei Dong, Haochuan Long, Zhen Liu, Yulu Guo, Yuji Xiao, Wenyan Liao

**Affiliations:** 1 The First Affiliated Hospital, Department of Gynaecology and Obstetrics, Hengyang Medical School, University of South China, Hengyang, Hunan, China; 2 The First Affiliated Hospital, Department of Hepatopancreatobiliary Surgery, Hengyang Medical School, University of South China, Hengyang, Hunan, China

**Keywords:** anti-phospholipid syndrome, bioinformatics analyses, immune infiltration, machine learning, nomogram, recurrent pregnancy loss, regulatory networks

## Abstract

**Background:**

Recurrent Pregnancy Loss (RPL) is significantly associated with Antiphospholipid Syndrome (APS), yet the shared pathogenic mechanism remains unclear. This study identifies potential hub genes for APS-related RPL, providing insights into its pathogenesis and potential diagnostic strategies.

**Methods:**

We retrieved APS and RPL datasets from the GEO database and performed differential expression analysis to identify differentially expressed genes. Thereafter, Gene Ontology (GO) analysis, pathway enrichment analysis, weighted gene co-expression network analysis (WGCNA), and protein-protein interaction (PPI) analysis were carried out. For hub gene screening, Least Absolute Shrinkage and Selection Operator (LASSO) regression, Gaussian mixture model (GMM), and RandomForest (RF) algorithms were applied to analyze the gene expression profiles of RPL. The CIBERSORT tool was utilized to assess the immune cell infiltration levels in samples from RPL and APS patients, while the “clusterProfiler” R package was employed to perform single-gene Gene Set Enrichment Analysis (GSEA) for each hub gene in both cohorts. Three hub genes were validated for diagnostic performance via receiver operating characteristic (ROC) curves, with a nomogram model subsequently constructed and its efficacy assessed using ROC and calibration curves. Additionally, the Comparative Toxicogenomics Database was used to explore the associations between these hub genes and pregnancy-related diseases. Finally, quantitative polymerase chain reaction (qPCR) and cell function experiments were conducted to validate the expression and functional characteristics of the hub genes.

**Results:**

In this study, RPL and APS datasets were obtained from Gene Expression Omnibus (GEO) database, 10 common differentially expressed genes(DEGs)were identified, including eight downregulated and two upregulated genes. The analysis of these shared DEGs indicated that the imbalance of immune system-associated cells and molecules might represent a common characteristic in the pathophysiological processes of both APS and RPL. Through machine learning and the construction of a nomogram, we identified NAA30, ARHGAP44, and SUGT1 as hub genes. Further experiments revealed that SUGT1 was downregulated in RPL with APS and could influence the biological behavior of trophoblast cells.

**Conclusion:**

The present study provides valuable insights into the molecular mechanisms underlying RPL with APS, while also identifying potential biomarkers and therapeutic targets for this disease.

## 1 Introduction

Clinically, Recurrent Pregnancy Loss (RPL) refers to two or more pregnancy losses happening before 20–24 weeks of gestation. This prevalent pathological condition poses a significant challenge to human reproductive health and stands as a major pregnancy complication, affecting roughly 2.5% of women during pregnancy (1). Several factors have been shown to exert an influence on RPL risk, including those related to endocrine function, immunity, infection, and genetics (2, 3). Nevertheless, approximately 50% of RPL cases have an unknown etiology (4), While the carriage of specific gene variants has been associated with elevated susceptibility to RPL, only a small number of these high-risk variants have been confirmed to contribute to the pathogenesis of RPL (5, 6). Consequently, it is of great importance to investigate both the diagnostic genes linked to RPL and the underlying mechanisms driving the disease’s pathogenesis.

Antiphospholipid-antibody syndrome (APS), a type of acquired thrombophilia, may lead to vascular thrombosis (venous or arterial) and/or pregnancy-related losses. A necessary factor for the onset of these clinical events is the persistent detection of antiphospholipid antibodies (APL) (7). Chorionic plate microthrombosis at the maternal-fetal interface can be caused by APS. This condition further contributes to various adverse perinatal outcomes, including recurrent miscarriage, placental dysfunction, preterm pre-eclampsia, fetal growth restriction, fetal distress, and stillbirth (8, 9). Research indicates that 5% to 20% of women of childbearing age have clinical signs of APS. The pregnancy loss rate for patients with positive APLs can reach 24% to 60% in the absence of proper treatment. APS is recognized as the most common etiological factor underlying immune-related RPL, with a prevalence rate of approximately 7%–25% among this patient population (10, 11). *In vivo*, multiple pathogenic mechanisms can trigger T cell activation and the concomitant production of cytokines in patients with APS. These pathological processes not only impair the normal regulatory capacity of the immune system but also disrupt immunological homeostasis (12, 13). Furthermore, APL exert inhibitory effects on the migration and invasion of chorionic villous cells, all of these effects can ultimately induce abortion (12, 14, 15). Taken together, these observations provide strong evidence supporting an association between APS and RPL. However, the underlying mechanisms remain insufficiently clarified, highlighting the necessity of investigating the shared pathophysiological processes and genetic characteristics of the two conditions.

**Figure 1:**
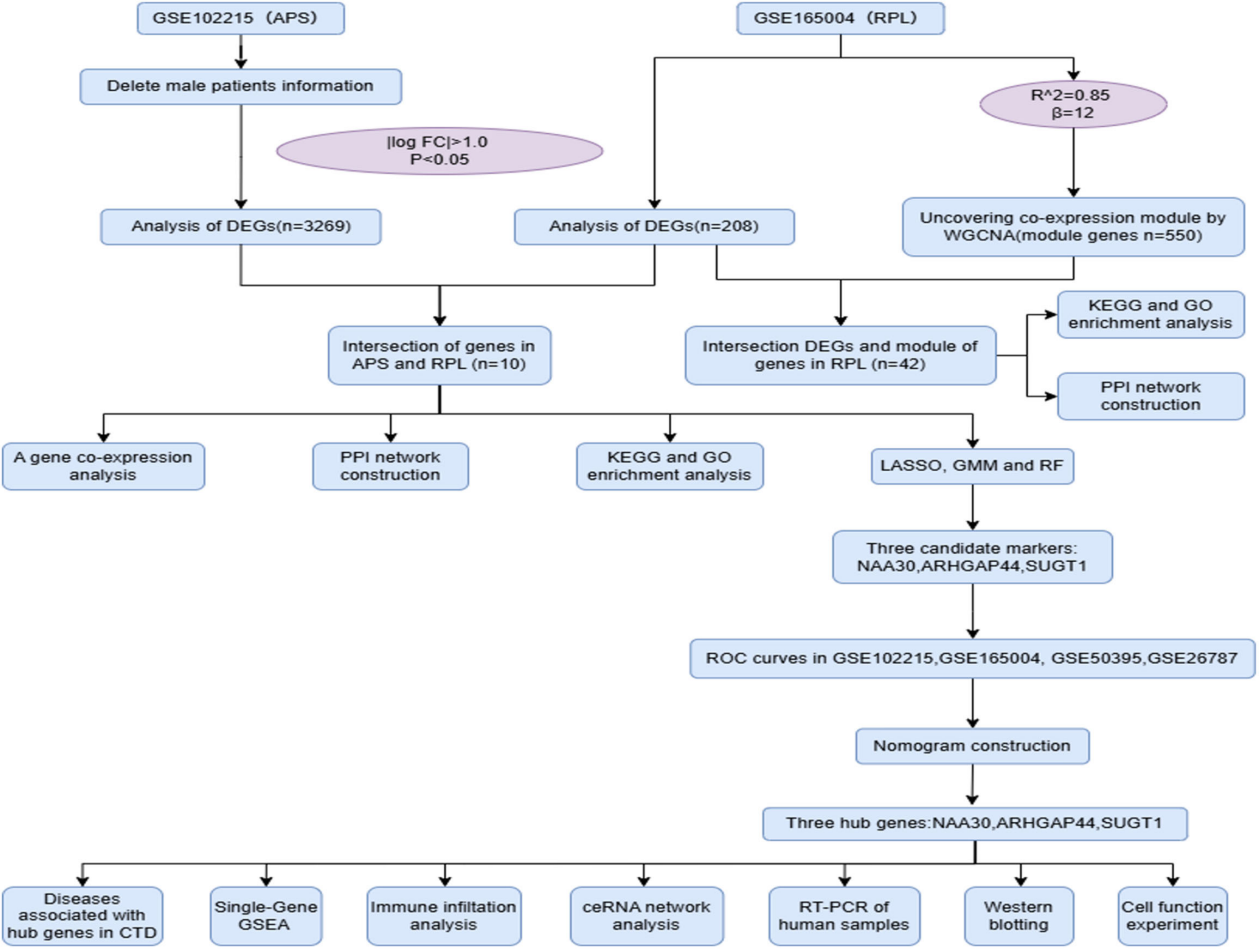
Flow chart of research.

To explore the shared pathogenesis of APS and RPL, we first screened public databases to identify common DEGs between these two disorders. Subsequently, these shared DEGs were utilized to construct diagnostic models and perform experimental validation. Notably, the findings of this work are expected to offer novel insights and research directions for exploring the biological mechanisms underlying RPLwith APS, thereby providing support for the development of targeted therapeutic strategies. The study workflow is illustrated in **Figure 1**.

## 2 Materials and methods

### 2.1 Acquisition and preprocessing of data

We conducted a search in the GEO database (https://www.ncbi.nlm.nih.gov/gds) for relevant gene expression datasets using the key terms “antiphospholipid syndrome” and “Recurrent pregnancy loss”. During the data screening process, specimens derived from male subjects and non-human samples were excluded to ensure the relevance and specificity of the data to our research focus. After completing the screening procedure, four datasets were ultimately retrieved from the GEO database, namely GSE102215 (which relies on the GPL16791 platform), GSE50395 (based on the GPL4133 platform), GSE165004 (utilizing the GPL16699 platform), and GSE26787 (also dependent on the GPL570 platform).

### 2.2 Identification of shared genes

For the GSE102215 dataset, after excluding samples from male patients, differential expression analysis was performed to identify DEGs between healthy control samples and APS samples using the “edgeR” R package. Here, DEGs were defined as genes satisfying the strict cutoff criteria: P < 0.05 and absolute value of log fold change (|logFC|) > 1.0. Regarding the GSE165004 dataset, the same cutoff thresholds (P < 0.05 and |logFC| > 1.0) were applied to determine DEGs.

To visualize the expression patterns of the identified DEGs, volcano plots and heatmaps were generated. Specifically, the “ggplot2” R package and “pheatmap” R package were used for local visualization, while the online platform (https://hiplot.cn/basic/volcano) was also employed for volcano plot construction. For the screening of common DEGs linked to both APS and RPL, the “dplyr” R package, “openxlsx” R package, and “VennDiagram” R package were jointly utilized to extract and summarize the overlapping DEGs across the relevant datasets.

### 2.3 Weighted gene co-expression network analysis-based module gene selection for RPL

The gene co-expression network was established by means of the WGCNA package, which supports the identification of potential links between gene networks and clinical parameters. In the GSE165004 dataset, the top 25% of genes with the highest expression variance were used for WGCNA. The soft‐thresholding power β was determined based on the scale‐free topology principle. We tested powers from 1 to 20, and selected the smallest β that achieved a scale‐free topology fit index R² ≥ 0.85. Accordingly, β=12 was chosen. The adjacency matrix was transformed into a topological overlap matrix (TOM) to reduce noise. Gene modules were identified by hierarchical clustering with a minimum module size of 30 genes. Module eigengenes were correlated with disease status, and the most significantly associated module was selected. Hub genes were screened with gene significance (GS) > 0.5 and module membership (MM) > 0.8.

### 2.4 Gene ontology and kyoto encyclopedia of genes and genomes databases based functional enrichment analysis

Functional enrichment analysis was conducted using the R package “clusterProfiler”, with the threshold set at P-value < 0.05. Notably, both GO enrichment analysis and KEGG enrichment analysis were performed twice throughout the research process.

### 2.5 Establishment of protein-protein interaction networks

Search Tool for the Retrieval of Interacting Genes (STRING; https://cn.string-db.org/) was employed to construct protein-protein interaction (PPI) networks for module genes and common DEGs, followed by visualization of these PPI networks via Cytoscape software.

### 2.6 Co-expression analysis of common DEGs

To further dissect the interrelationships among common DEGs, the GeneMANIA online tool (http://genemania.org/) was utilized for co-expression network construction. This database employs large-scale genomic and proteomic data to generate co-expression networks of identified common genes, allowing the recognition of functionally linked genes and their weighting according to expected values.

### 2.7 Machine learning

To identify candidate genes of RPL with APS, we conducted further screening by means of three machine learning algorithms, including least absolute shrinkage and selection operator (LASSO) regression, Gaussian Mixture Model (GMM), and Random Forest(RF). To guarantee the reproducibility of these algorithms, a fixed seed (66666) was set for both disease cohorts. For LASSO regression (R package: “glmnet”), the 10 candidate genes were used as predictors, and disease status was defined as the binary response variable with “family =binomial”. L1 regularization was applied for feature selection. Five-fold cross-validation was used to avoid overfitting, and λ.min (minimum cross-validated MSE) was selected as the optimal penalty parameter. Genes with non-zero coefficients at λ.min were retained as candidate biomarkers. GMM (R packages: “mclust”, “pROC”, “SimDesign”) explored gene expression distributions via multiple Gaussian fits to reveal biological information. Random Forest (R package: “randomForest”) constructed an integrated decision tree forest for outcome prediction. Algorithm outputs were intersected using a Venn diagram, with overlapping genes defined as hub genes.

### 2.8 Identification and validation of hub genes

To assess the diagnostic utility of candidate hub genes for RPL and APS, receiver operating characteristic (ROC) curves were generated using the “pROC” R package, with the area under the curve (AUC) calculated for each. An ideal diagnostic value was defined as AUC > 0.6. Two additional datasets (RPL: GSE26787; APS: GSE50395) were utilized for external validation.

### 2.9 Hub gene interactions with diseases

To investigate candidate diagnostic gene-disease relationships, inference scores and reference counts for these genes and their associated diseases were analyzed using Comparative Toxicogenomics Database (CTD) (http://ctdbase.org/), with results visualized as histograms.

### 2.10 Nomogram construction and evaluation

Nomogram construction holds value for the clinical diagnosis of RPL with APS. A nomogram was developed using candidate hub genes via the “rms” R package, where “Points” indicates the score of each candidate hub gene and “Total Points” represents the sum of these individual gene scores. Nomogram performance verification included C-index calculation, discrimination evaluation (1000-resample bootstrap), and calibration curve plotting to compare ideal vs. actual diagnostic rates. The model’s diagnostic performance was assessed via ROC curves, with AUC and 95% CI generated; AUC > 0.7 was deemed excellent diagnostic efficacy.

### 2.11 GSEA implementation for a single hub gene

Following the acquisition of diagnostic genes, we implemented single-gene Gene Set Enrichment Analysis (GSEA) for each diagnostic gene across the two groups with the aid of the R package “clusterProfiler”. This analytical approach enabled us to compare differences in biological signaling pathways between the disease group and healthy control group. Gene sets were downloaded from the MSigDB database (accession: c5.go.bp.v7.5.1.entrez.gmt), and the Enrichplot tool was utilized to visualize the top 5 activated and inhibited pathways for each gene in the two disease groups.

### 2.12 Assessment of immune cell infiltration

To estimate immune cell infiltration from gene expression profiles, we employed CIBERSORT, a specialized analytical tool. Using this platform, we assessed immune cell proportions in the disease groups. Bar graphs visualized immune cell proportional abundances, and violin plots compared these proportions between APS/control and RPL/control groups. Correlation analyses explored associations between immune cell subsets and hub genes, with results visualized using the “corplot”, “vioplot”, and “ggpubr” R packages.

### 2.13 Prediction of miRNAs regulating three hub genes

To further explore the regulatory associations between the three hub genes and their corresponding miRNAs, we retrieved miRNA candidates targeting each hub gene from the miRWalk database. Subsequently, based on the identified interactions between these hub genes and miRNAs, we constructed an integrated miRNA-mRNA (hub gene) regulatory network using Cytoscape software.

### 2.14 Human samples collection

Between June 2023 and June 2025, this study enrolled 15 women (25–39 years old) withRPL(defined as two or more spontaneous abortions) and 15 fertile women (24–38 years old) with one or more live births and no history of spontaneous abortion from the Department of Obstetrics and Gynecology, the First Affiliated Hospital of University of South China. For RPL patient inclusion, only those diagnosed in accordance with the Sydney modification of the APS classification criteria were eligible (16). General exclusion criteria comprised embryonic chromosomal abnormalities, abnormal parental karyotypes, reproductive tract anatomical anomalies, endocrine disorders, and infectious etiologies of abortion. Decidual tissues and villous tissues were obtained from induced abortions carried out during the 6–10 gestational weeks, and written informed consent for sample utilization was provided by each participant. Following collection, all specimens were promptly snap-frozen in liquid nitrogen and preserved at -80 °C until subsequent laboratory analysis. This study was approved by the Research Ethics Committee of the First Affiliated Hospital of University of South China (No.2023ll0518001) and conducted in strict compliance with the Declaration of Helsinki. Baseline characteristics of all women were recorded (**Supplementary Table 1**).

### 2.15 Total RNA extraction and quantitative real-time PCR

Total RNA was extracted from samples following the TRIzol Reagent (Invitrogen) protocol, followed by reverse transcription into cDNA using the PrimeScript™ RT reagent kit with gDNA Eraser (Takara, Japan). qRT-PCR was performed with the SYBR Premix Ex Taq kit II (Takara, Japan). The relative mRNA expression levels were calculated using the 2^-ΔΔ^CT method and normalized to GAPDH as the internal reference gene. The primers were designed and synthesized by Sangon Biotech Co., Ltd(China), and the primer sequences are shown in **Supplementary Table 2**.

### 2.16 Western blot assay

Total proteins were extracted from the samples using RIPA lysis buffer purchased from Beyotime Biotechnology (Shanghai, China). Subsequently, SDS-PAGE loading buffer was added to the protein mixtures, which were then heated at 100 °C for 5 minutes to ensure full protein denaturation. Denatured protein samples underwent separation via sodium dodecyl sulphate-polyacrylamide gel electrophoresis (SDS-PAGE), followed by electrotransfer onto polyvinylidene fluoride (PVDF) membranes.Post-transfer, the PVDF membranes were blocked with 5% non-fat milk for 1 hour at room temperature to eliminate non-specific antibody binding. The membranes were then subjected to overnight incubation with primary antibodies at 4 °C, followed by treatment with horseradish peroxidase (HRP)-conjugated secondary antibodies (Beyotime, Shanghai, China). The primary antibodies employed were SUGT1 antibody (1:2000 dilution, Proteintech, China) and β-tubulin antibody (1:1000 dilution, Cell Signaling Technology, USA). Protein bands were visualized using an enhanced chemiluminescence (ECL) detection system (Quickchemi 5200, Monad, China). Quantitative analysis of protein expression was performed by digital densitometry of the protein bands using ImageJ software (National Institutes of Health, USA).

### 2.17 Cell culture and treatment

HTR-8/SVneo trophoblast cells were obtained from Shanghai Zhong Qiao Xin Zhou Biotechnology Co., Ltd. (Shanghai, China). RPMI 1640 medium (Gibco, Carlsbad, CA, USA) supplemented with 10% fetal bovine serum (FBS, Gibco) was used for seeding HTR-8/SVneo cells. Cells were cultured under the conditions of 37°C and 5% CO_2_. Small interfering RNAs (siRNAs) specific to SUGT1, including siSUGT1–1 and siSUGT1-2, as well as their respective negative control (si-NC), were purchased from Ribobio Co., Ltd. (Guangzhou, China). SUGT1 full length cDNA was cloned into pcDNA3.1 vector. Transfections into cells were performed using Lipofectamine 2000 (Invitrogen, Thermo Fisher Scientific, USA) according to the manufacturer’s instruction.

### 2.18 EDU assay

Cell proliferation capacity was evaluated via the EdU cell proliferation assay kit (Beyotime Biotechnology, Shanghai, China). HTR-8/SVneo cells following transfection were plated into 24-well culture plates and incubated for a 24-hour period. Subsequently, the cells were treated with 10 μM EdU solution for 2 hours, after which they were fixed using 4% paraformaldehyde. Following the above treatments, the cells were subjected to Hoechst 33342 staining for the purpose of nuclear visualization. Ultimately, EdU-positive cells were visualized via an Olympus FSX100 fluorescence microscope (Olympus Corporation, Tokyo, Japan). Quantitative analysis of the acquired data was performed utilizing Image J software.The ratio of EdU-positive proliferating cells was determined as the proportion of EdU-positive cells relative to Hoechst-positive cells (representing total nuclei).

### 2.19 Cell migration and invasion analysis

To determine the migration and invasion potentials of HTR-8/SVneo cells, scratch assay and transwell invasion assay were utilized. For migration detection, transfected cells with si-SUGT1, SUGT1, or their negative controls were plated in 6-well plates and grown to 80% confluency. A pipette tip was applied perpendicularly to the plate base to make a consistent scratch across the cell monolayer. Cell migration was recorded via photography at 0 h, 24 h, with scratch gap lengths measured quantitatively using ImageJ software. For the analysis of cell invasion, HTR-8/SVneo cells following transfection were plated into matrigel-precoated 24-well Transwell inserts (Transwell, Corning Incorporated, NY, USA; matrigel, BD Biosciences, New York, USA). Following 24 h of incubation, invasive cells attached to the membrane’s lower surface were fixed in methanol for 30 min and stained with 0.1% crystal violet for another 30 min. Stained cells were counted under a microscope to compute the relative invasion rate.

### 2.20 Statistical analysis

All experiments were independently repeated at least three times. Statistical evaluations were conducted using GraphPad Prism 8 (GraphPad Software, Inc., La Jolla, CA, USA) and SPSS 19.0 (IBM, Armonk, NY, USA). Differences between two groups were assessed by Student’s t-test or Mann-Whitney U test, while one-way analysis of variance (ANOVA) was employed for comparisons among multiple groups. A P-value < 0.05 was considered statistically significant.

## 3 Results

### 3.1 Information extraction from GEO datasets

Four Gene Expression Omnibus (GEO) datasets (GSE102215, GSE50395, GSE165004, and GSE26787) were included in the analysis. All relevant characteristics of these datasets, ranging from GSE accession numbers and detection platforms to associated diseases, sample sizes, source types and grouping criteria, are summarized in **Table 1**. Among them, GSE102215 and GSE165004 were combined for differential expressed gene (DEG) analysis, while GSE50395 and GSE26787 were paired to validate the diagnostic efficiency of hub genes.

**Table 1:** Summary of the four GEO datasets involving APS and RPL patients.

ID	GSE number	Platform	Disease	Samples	Source types	Grope
1	GSE102215	GPL16791	APS	6 patients and 6 controls	Peripheral venous blood	Discovery cohort
2	GSE50395	GPL4133	APS	3 patients and 3 controls	Peripheral venous blood	Validation cohort
3	GSE165004	GPL16699	RPL	24 patients and 24 controls	Endometrium	Discovery cohort
4	GSE26787	GPL570	RPL	5 patients and 5 controls	Endometrium	Validation cohort

### 3.2 Screening of common DEGs

**Figure 2:**
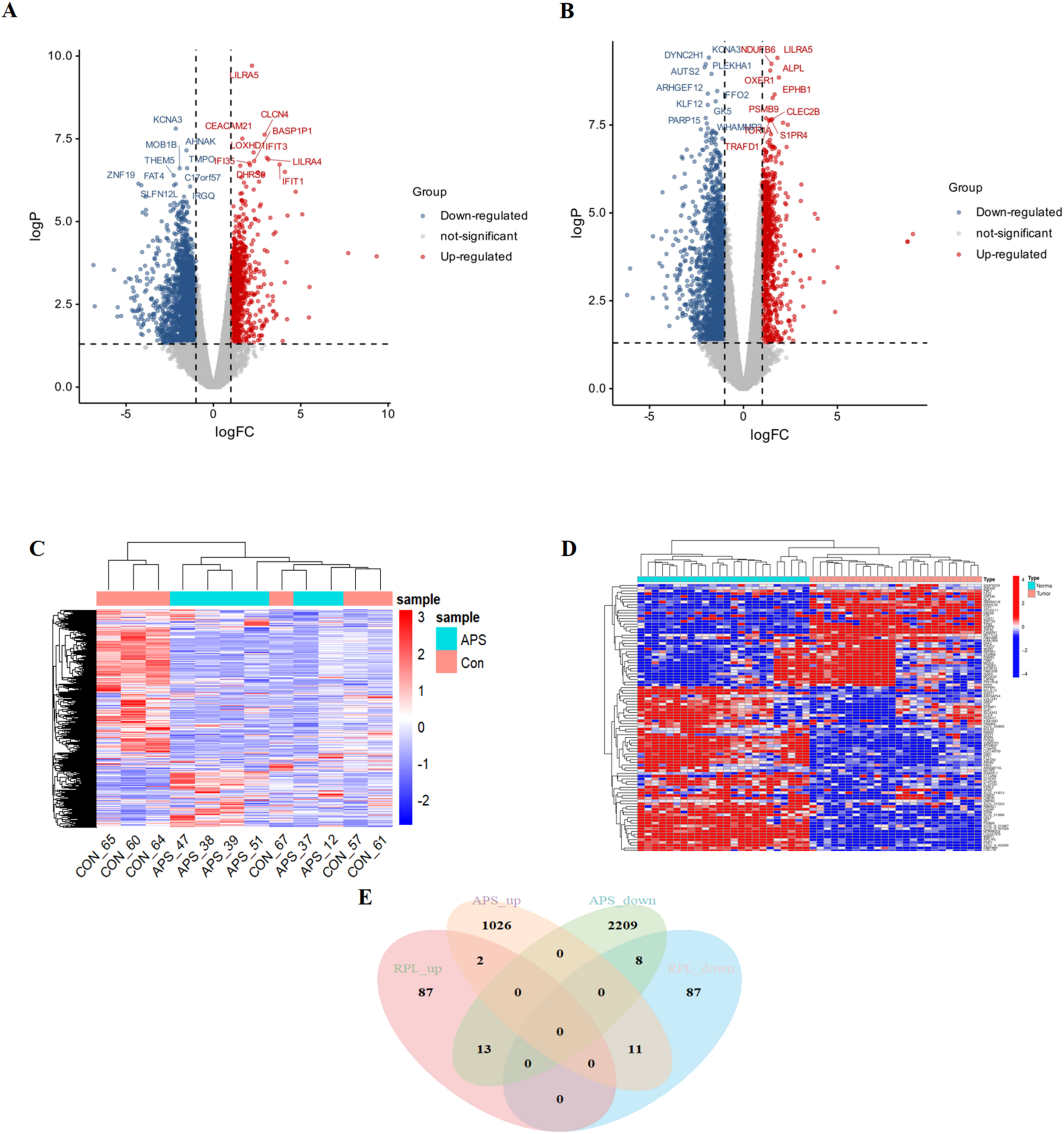
Screening of DEGs in APS and RPL. **(A)** Volcano plot of APS-related DEGs. **(B)** Volcano plot of RPL-related DEGs. **(C)** Heatmap of upregulated and downregulated DEGs in APS. **(D)** Heatmap of upregulated and downregulated DEGs in RPL. **(E)** Venn diagram showing common DEGs between APS and RPL.

Differentially expressed genes (DEGs) related to RPL and APS were retrieved from the GSE165004 and GSE102215 datasets, respectively. For the APS cohort (GSE102215), 1, 039 upregulated DEGs and 2, 230 downregulated DEGs were screened out, whereas the RPL dataset (GSE165004) yielded 102 upregulated DEGs and 106 downregulated DEGs (**Figures 2A, B**). Expression profiles of these DEGs in each dataset were visualized via heatmaps (**Figures 2C, D**). Shared DEGs between the two datasets were screened using Venn diagram intersection analysis (**Figure 2E**). Subsequent to excluding genes with contradictory expression tendencies, a total of 10 common DEGs with consistent expression patterns were retained. This set included eight universally downregulated genes and two consistently upregulated genes (**Table 2**).

**Table 2:** The gene expression levels of 10 common DEGs in APS and RPL.

Gene samples	GSE102215		GSE165004		Up/Down
	logFC	P Value	logFC	P Value	
ALPL	1.877956661	0.00000785	1.247411679	0.0135453	Up regulated
RAB11FIP1	1.258565592	0.00312412	1.261834444	0.0030805	Up regulated
CLIC4	-1.682541524	0.0000512	-1.213978231	0.0025839	Down regulated
ATP10A	-1.75401885	0.00019065	-1.518352033	0.0370973	Down regulated
CD2AP	-1.414965144	0.00198417	-1.234494773	9.576E-15	Down regulated
MATN2	-1.606107685	0.0049643	-1.543911114	0.0138512	Down regulated
ARHGAP44	-2.261086037	0.00791129	-1.619820611	0.0003246	Down regulated
TNFRSF25	-1.460955527	0.00865918	-1.408638522	0.0226527	Down regulated
NAA30	-1.173647095	0.0104234	-1.234494773	0.0003845	Down regulated
SUGT1	-1.843448553	0.01473609	-1.102767714	2.04E-10	Down regulated

### 3.3 PPI Network construction and enrichment analysis of common DEGs

**Figure 3:**
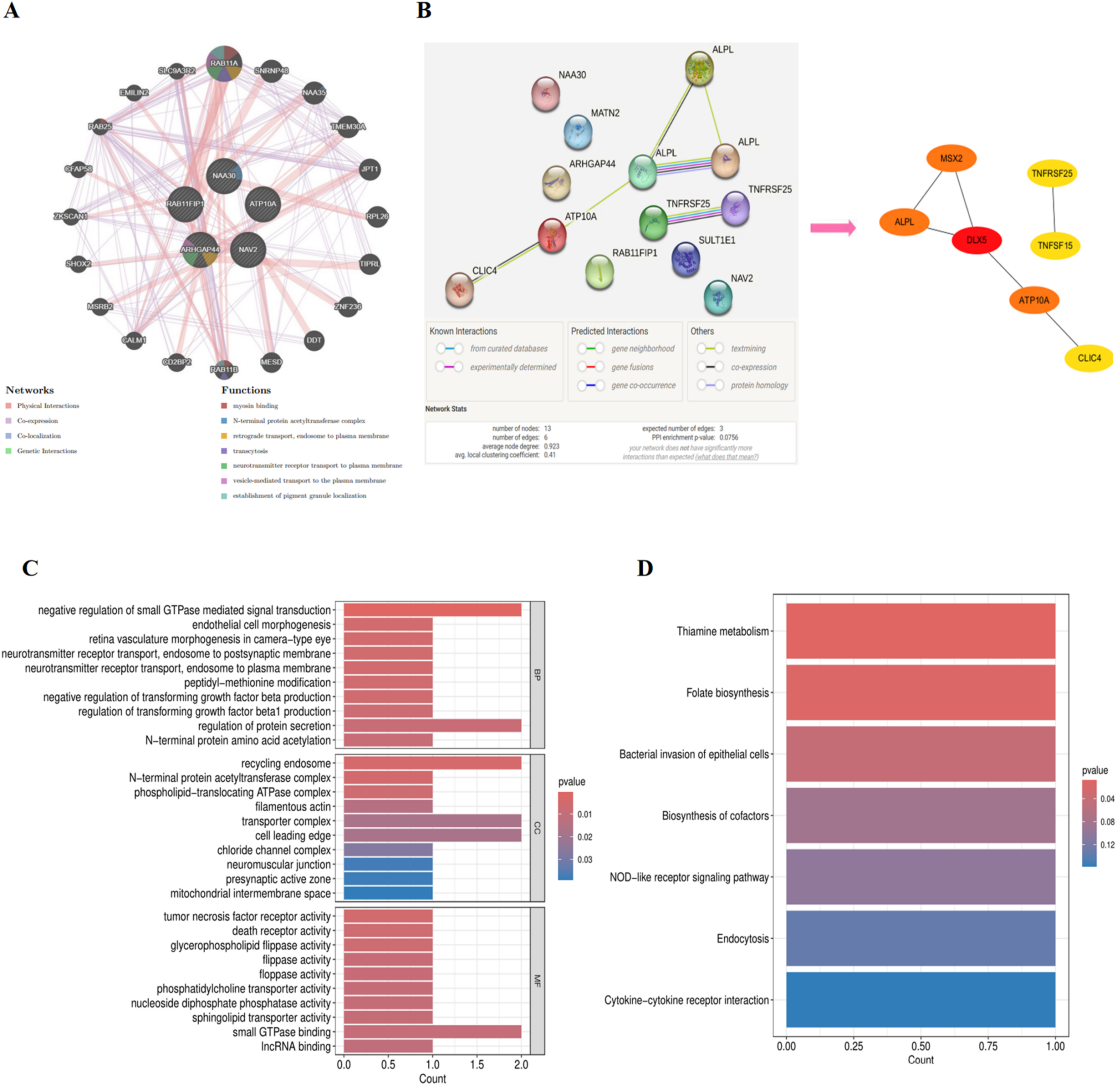
Analysis of common DEGs between APS and RPL. **(A)** GeneMANIA analysis of common DEGs and their co-expressed genes. **(B)** Protein-protein interaction (PPI) network of the common DEGs. **(C)**GO Enrichment Analysis of Common DEGs. **(D)** KEGG pathway enrichment analysis of common DEGs.

To explore the biological functions of these common DEGs, a refined gene interaction network was constructed via GeneMANIA, showing 99.92% co-expression and 0.08% genetic interactions. Network analysis identified 20 genes associated with common DEGs, with functional annotation suggested potential involvement in processes such as N-terminal protein acetyltransferase complex formation, retrograde transport from endosome to plasma membrane, transcytosis, vesicle-mediated transport to the plasma membrane, and so on (**Figure 3A**). Additionally, PPI network analysis indicated DLX5, ALPL, MSX2, and ATP10A had high connectivity with other nodes (**Figure 3B**). GO and KEGG pathway analyses implied potential links between these common DEGs and diverse biological functions and signaling pathways (**Figures 3C, D**). GO annotations were classified into three major categories: cellular component, molecular function, and biological process. Within the cellular component category, recycling endosomes emerged as the most highly represented term. Tumor necrosis factor receptor activity ranked as the top enriched GO term in the molecular function category. Furthermore, these common DEGs participate in multiple biological processes, such as negative regulation of small GTPase-mediated signal transduction, endothelial cell morphogenesis, and peptidyl-methionine modification. Notably, these common DEGs might be closely associated with numerous immune-related pathways, including NOD-like receptor signaling pathway, cytokine-cytokine receptor interaction and so on.

### 3.4 Co-expression modules in RPL

As an autoimmune disorder, APS is pathophysiologically linked to *in vivo* immune homeostasis perturbations. To clarify the association between RPL and the systemic immune microenvironment, we analyzed RPL-related key genes.

**Figure 4:**
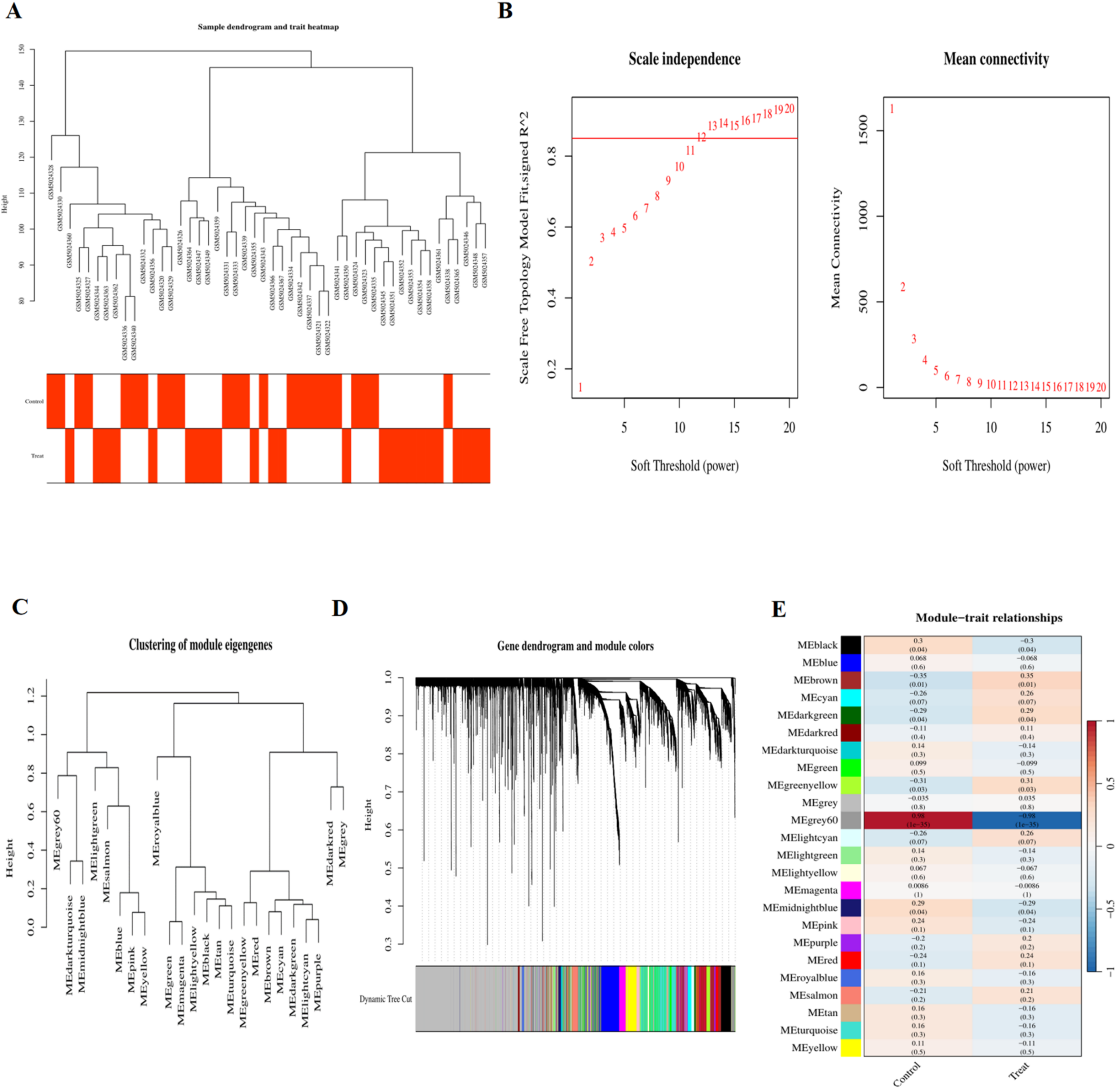
WGCNA of RPL. **(A)** Sample clustering of RPL. Three distinct clusters were identified, all of which were included for further analysis. **(B)** Selection of optimal thresholds. **(C)** Threshold set to 0.85 to merge similar modules in the cluster tree. **(D)** Modules generated by aggregating highly correlated genes, with different colors representing distinct modules. **(E)** Heatmap of module-trait relationships in RPL. Each cell shows the corresponding correlation coefficient and P-value.

In the GSE165004 dataset, genes with the top 25% variability were selected as key RPL genes for WGCNA. All samples clustered well without outlier exclusion (**Figure 4A**). The optimal soft-power value for GSE165004 was β=12 (R²=0.85) (**Figures 4B, C**), identifying 24 co-expression modules. Module-clinical disease correlation analysis showed the brown module had the strongest positive association (r=0.35, P = 0.01), while the grey60 module had the most significant negative correlation (r=-0.98, P = 1e-35) (**Figures 4D, E**). Genes in RPL-related co-expression modules are listed in **Supplementary Table 3**.

### 3.5 Screening and functional analysis of key genes in RPL

**Figure 5:**
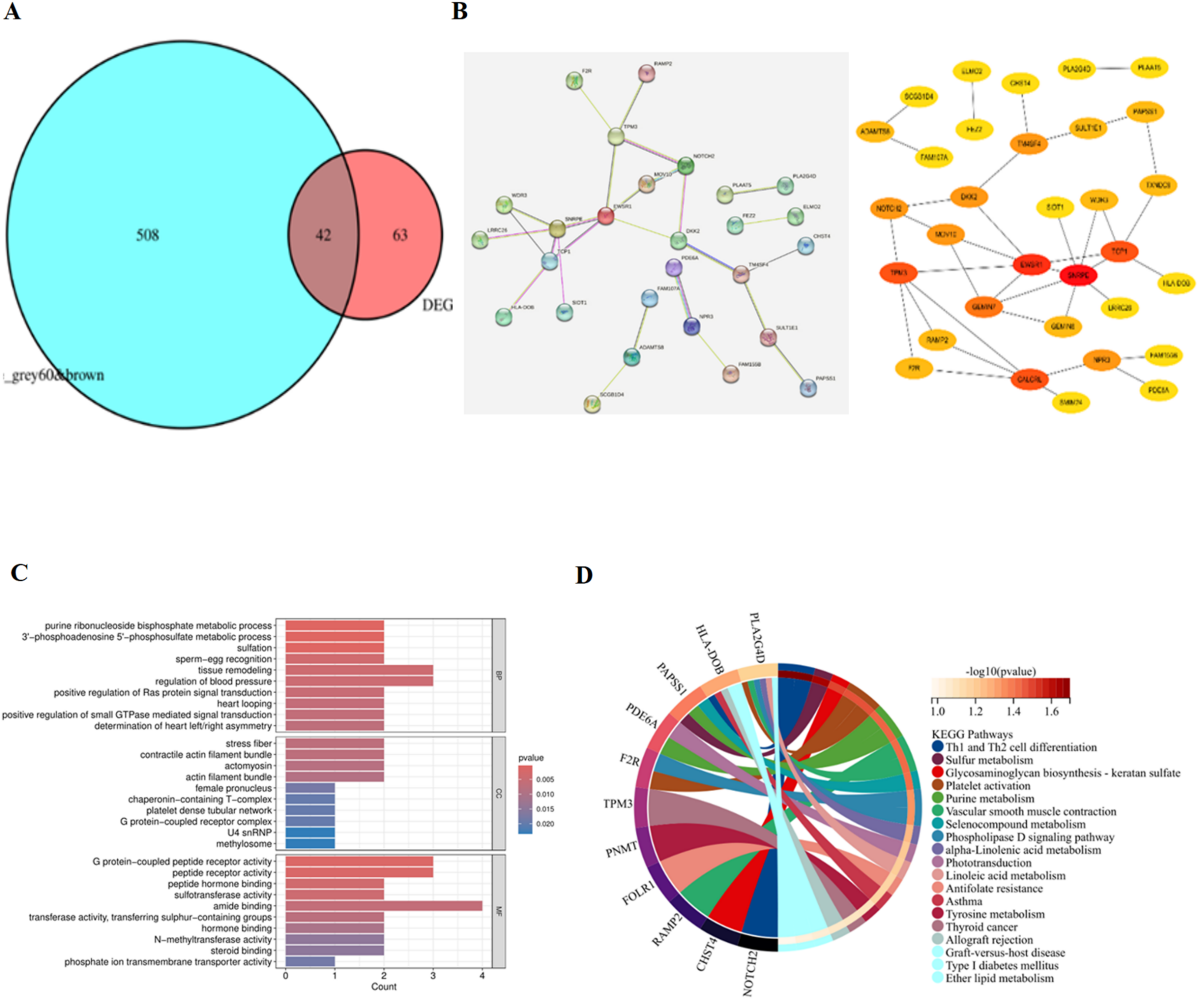
Analysis of key genes in RPL. **(A)** Key genes defined as overlapping DEGs and module genes. **(B)** PPI network of the key genes. **(C)** Functional enrichment clustering analysis of key genes based on GO terms (biological process [BP], cellular component [CC], molecular function [MF]). **(D)** KEGG pathway analysis of the key genes.

We identified 42 genes overlapping between the RPL-related DEG set and module gene clusters as key genes (**Figure 5A**, **Supplementary Table 4**), and these genes were closely correlated with the pathogenesis of RPL. Additionally, PPI network analysis revealed tight interactions among these key genes (**Figure 5B**). To explore the functions of these key genes, we performed robust GO and KEGG enrichment analyses using this larger and more reliable gene set. From the perspective of biological processes, the key genes were predominantly implicated in processes such as purine ribonucleoside bisphosphate metabolic process, sperm-egg recognition, and positive regulation of Ras protein signal transduction. In terms of cellular components, they were associated with stress fibers, G protein-coupled receptor complex, and so on. At the molecular function level, their associations included G protein-coupled peptide receptor activity, peptide receptor activity, phosphate ion transmembrane transporter activity, and more (**Figure 5C**). KEGG analysis indicated that the key genes markedly enriched in several pathways, including Th1 and Th2 cell differentiation, linoleic acid metabolism, antifolate resistance, tyrosine metabolism, and others (**Figure 5D**).

### 3.6 Screening of hub genes based on machine learning

**Figure 6:**
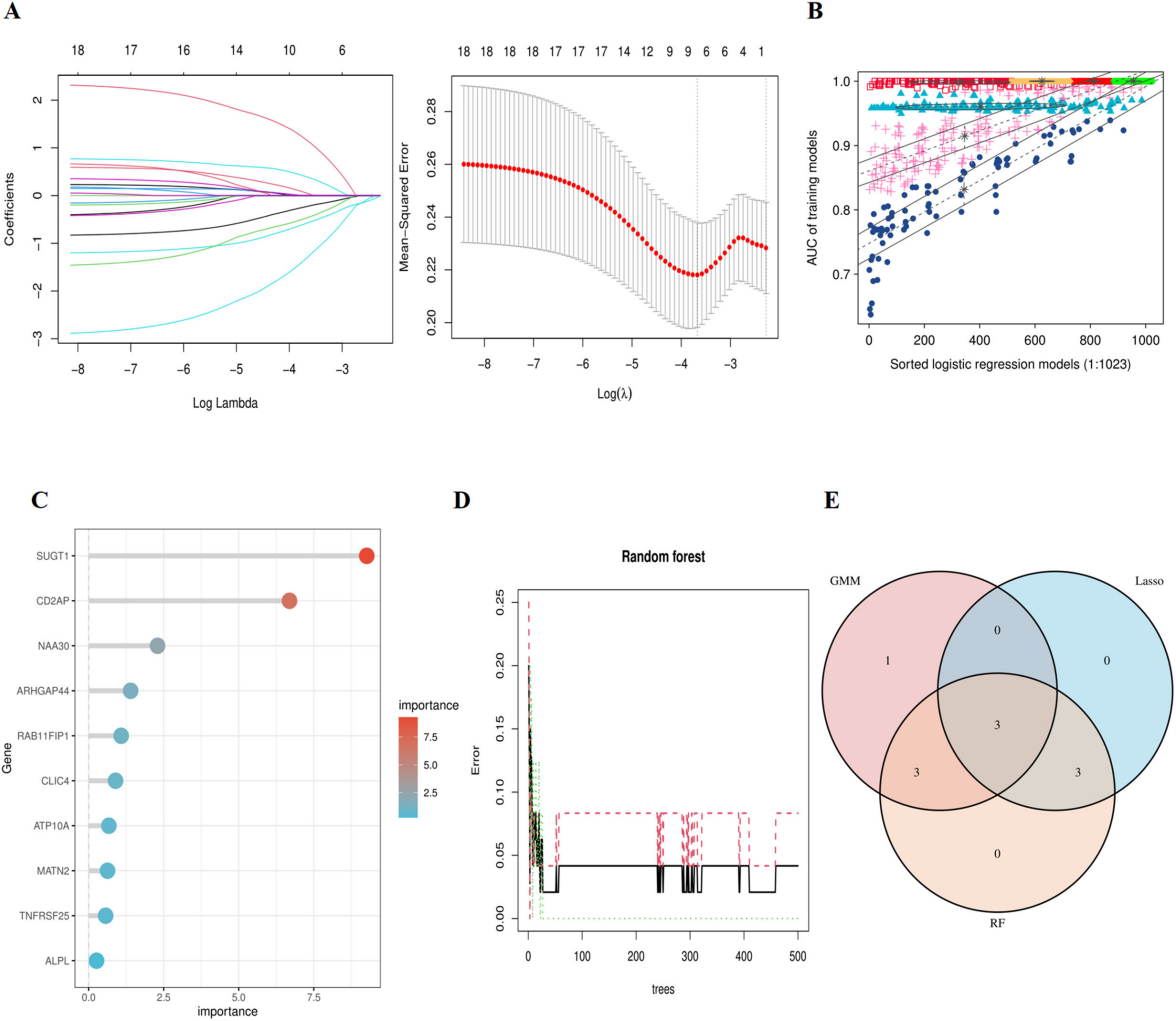
Screening of hub genes based on machine learning. **(A)** Coefficient profile plot of the LASSO model showing final parameter λ (lambda) selection. **(B)** Variable selection in the GMM model (n=7). **(C)** Top 10 genes from the RF analysis results. **(D)** Important features selected via the RF algorithm (n=9). **(E)** Venn diagram of results from the three machine learning models.

To further screen optimal candidate diagnostic genes that contribute significantly to distinguishing the disease group from the control group, three algorithms (LASSO, GMM, RF) were used based on 10 common genes from DEGs results. In the RPL group, LASSO (λ=0.01132778, **Figure 6A**) identified 6 genes with non-zero coefficients; GMM pinpointed 7 genes with the minimum error (**Figure 6B**); RF algorithm, following rigorous feature selection procedures, first identified the top 10 genes, and subsequent refinement based on RF importance analysis selected the top 9 genes with an importance score exceeding 0.5 (**Figures 6C, D**). The Venn diagram method intersected these three results, identifying three hub genes: NAA30, ARHGAP44, and SUGT1 (**Figure 6E**).

### 3.7 Validation of hub genes

**Figure 7:**
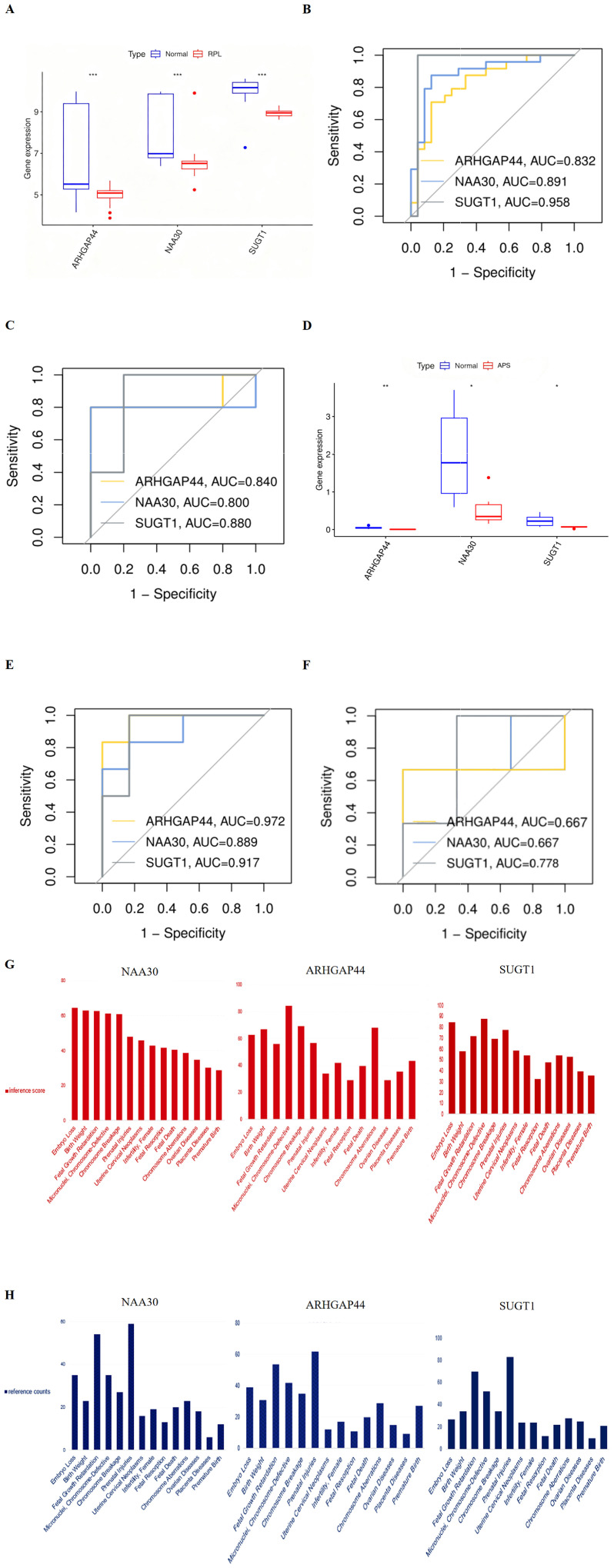
Validation of hub genes. **(A)** Differential expression of candidate diagnostic genes in the training group for RPL. **(B)** ROC curve of candidate diagnostic genes in GSE165004. **(C)** ROC curve of candidate diagnostic genes in GSE26787. **(D)** Differential expression of candidate diagnostic genes in the training group for APS. **(E)** ROC curve of candidate diagnostic genes in GSE102215. **(F)** ROC curve of candidate diagnostic genes in GSE50395. **(G)** Inference score between hub genes and embryo Loss, birth Weight, fetal growth retardation, micronuclei chromosome-defective, chromosome Breakage, prenatal Injuries, uterine cervical neoplasms, infertility female, fetal resorption, fetal death, chromosome aberrations, ovarian diseases, placenta diseases, premature birth in CTD. **(H)** Reference counts between hub genes and the aforementioned CTD-annotated phenotypes.(*P< 0.05, **P< 0.01, ***P< 0.001).

To gain a more precise insight into the association between RPL and APS, we evaluated the predictive and discriminatory capabilities of hub genes by analyzing the expression patterns of three hub genes, alongside conducting ROC curve analysis.First, we analyzed the expression levels of these genes in RPL and APS using two discovery cohorts. As presented in **Figures 7A, D**, NAA30, ARHGAP44, and SUGT1 exhibited significantly lower expression in both RPL and APS groups (P < 0.0001).To validate the reliability of these candidate diagnostic genes, we utilized datasets GSE165004 and GSE26787 to assess their diagnostic efficacy for RPL, and datasets GSE102215 and GSE50395 for APS. Collectively, all three genes demonstrated favorable diagnostic performance (**Figures 7B, C, E, F**). Additionally, analysis using the CTD revealed an association between these hub genes and adverse pregnancy outcomes in women (**Figures 7G, H**).

To further explore the potential molecular mechanisms of the three hub genes in RPL and APS, single-gene GSEA analyses were performed for the two datasets separately. The top 5 upregulated and downregulated signaling pathways were visualized according to enrichment scores. As shown in **Supplementary Figure 1**, the three hub genes were collectively enriched in multiple metabolic pathways, including arginine and proline metabolism, alpha-linolenic acid metabolism and linoleic acid metabolism. In addition, these genes were closely involved in inflammation-related signaling pathways, which further explained the underlying association between immune disturbance and disease pathogenesis in RPL and APS.

### 3.8 Immune infiltration analysis of hubgenes

**Figure 8:**
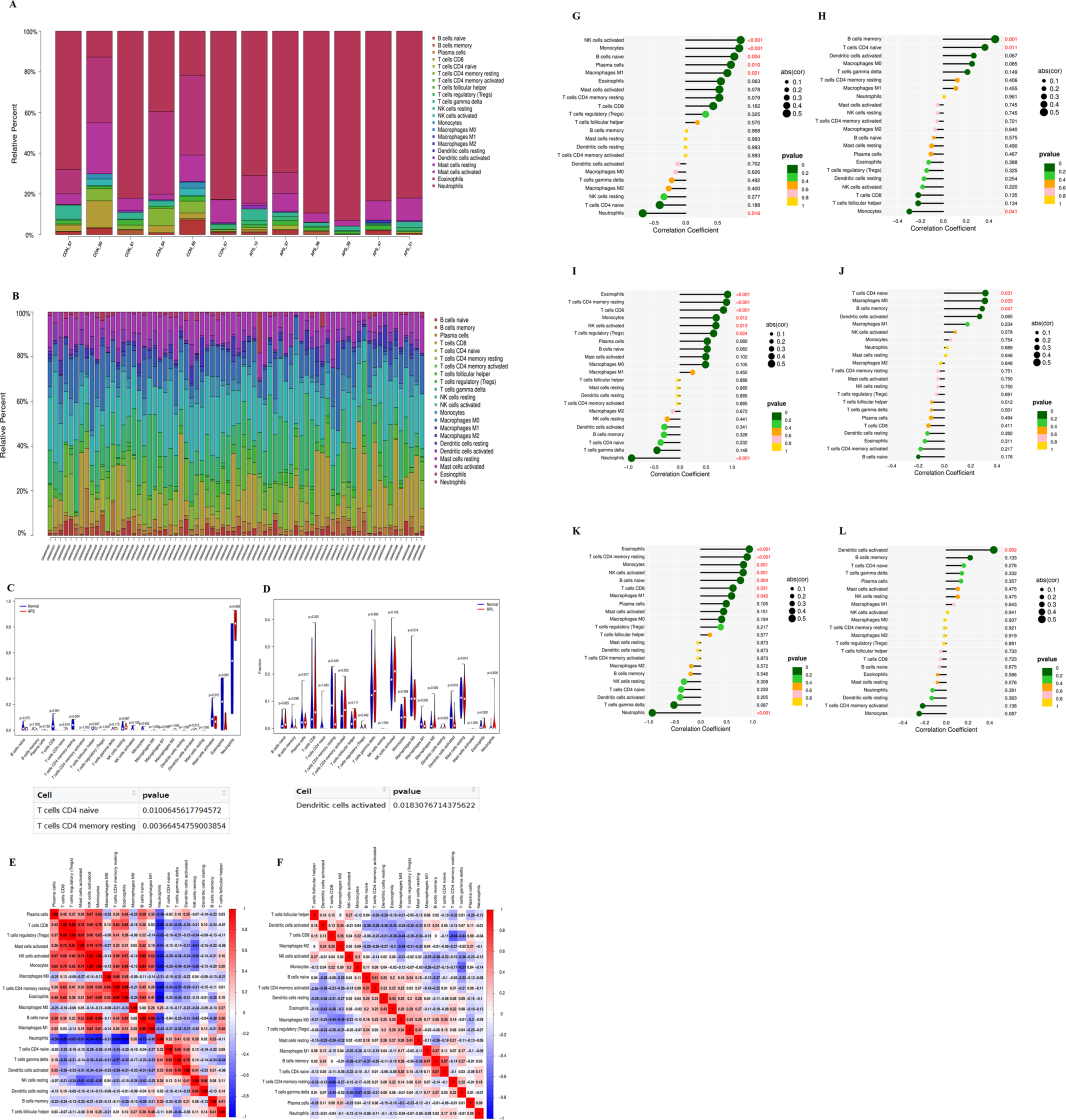
APS and RPL immune cell composition. **(A)** Stacked bar chart showing infiltrating immune cells in the APS cohort. **(B)** Stacked bar chart showing infiltrating immune cells in the RPL cohort. **(C)** Violin plot indicating immune cell types with significant differences in the APS cohort. **(D)** Violin plot indicating immune cell types with significant differences in the RPL cohort. **(E)** Correlation heatmap of 22 immune cell types in APS. **(F)** Correlation heatmap of 22 immune cell types in RPL. **(G, H)** Correlation analysis between ARHGAP44 and immune cells in APS **(G)** and RPL **(H)**. **(H-J)** Correlation analysis between NAA30 and immune cells in APS **(I)** and RPL **(J)**. **(K, L)** Correlation analysis between SUGT1 and immune cells in APS **(K)** and RPL **(L)**.

Given the prominent immune response characteristics of RPL and APS, we first analyzed the abundance of immune cells across different groups using CIBERSORT. The proportional distribution of 22 immune cell subsets in each group was visualized as bar plots, which clearly revealed potential differences in the proportions of neutrophils and T cell populations between the APS (**Figure 8A**) and RPL (**Figure 8B**) groups. As illustrated in the violin plots, APS samples showed an increase in naive CD4^+^ T cells and a decrease in resting memory CD4^+^ T cells (**Figure 8C**), while RPL samples exhibited reduced levels of activated dendritic cells (**Figure 8D**). Moreover, **Figures 8E, F** illustrate the correlations among individual immune cell types. Notably, different immune cells interact with each other and are closely interconnected. Of these immune cell populations, neutrophils display a markedly negative correlation with other immune cell types in APS.

To further explore the functional relevance of the identified biomarkers, we subsequently investigated their correlations with immune cell contents. In the APS group, ARHGAP44 was positively correlated with naive B cells, M1 macrophages, monocytes, activated NK cells, and plasma cells, while negatively correlated with neutrophils (**Figure 8G**, **Supplementary Figure 2A**); NAA30 showed positive correlations with eosinophils, monocytes, activated NK cells, resting memory CD4^+^ T cells, CD8^+^ T cells, and regulatory T cells (Tregs), and a negative correlation with neutrophils (**Figure 8I**, **Supplementary Figure 2C**); SUGT1 was positively associated with naive B cells, M1 macrophages, monocytes, activated NK cells, eosinophils, resting memory CD4^+^ T cells, and CD8^+^ T cells, and negatively correlated with neutrophils (**Figure 8K**, **Supplementary Figure 2E**). In the RPL group, ARHGAP44 exhibited positive correlations with memory B cells and naive CD4^+^ T cells, and a negative correlation with monocytes (**Figure 8H**, **Supplementary Figure 2B**); NAA30 was positively correlated with memory B cells, M0 macrophages, and naive CD4^+^ T cells (**Figure 8J**, **Supplementary Figure 2D**);SUGT1 displayed a positive association with activated dendritic cells.(**Figure 8L**, **Supplementary Figure 2F**).

### 3.9 Nomogram of hub genes in RPL

**Figure 9:**
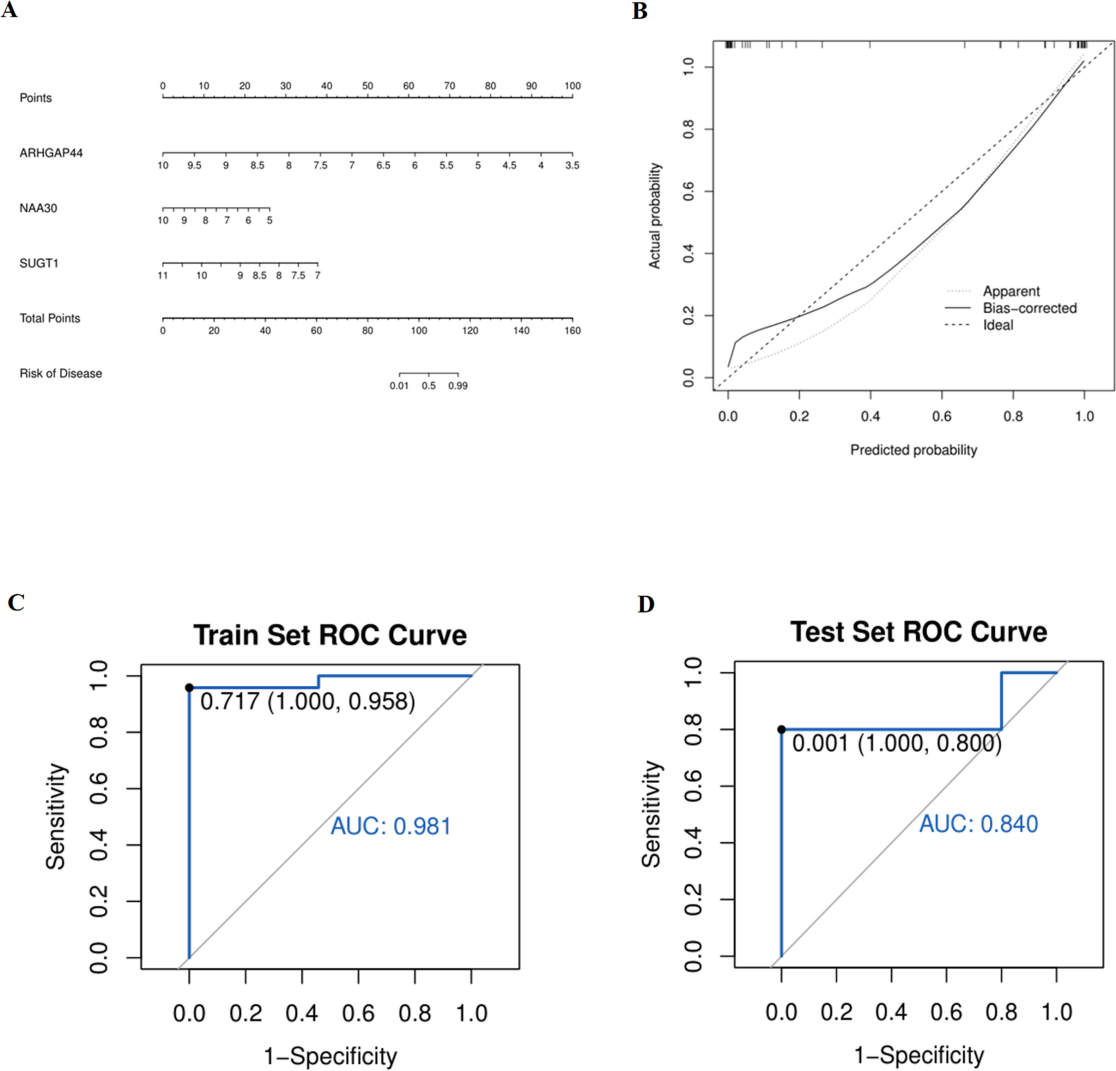
Nomogram of hub genes in RPL. **(A)** The nomogram for diagnosing RPL with APS. **(B)** Calibration curve plot for the nomogram. **(C)** The ROC curve of RPL nomogram model in GSE165004. **(D)** The ROC curve of RPL nomogram model in GSE26787.

To achieve a more precise identification of hub genes specific to RPL, a nomogram model was built with the machine learning-derived genes NAA30, ARHGAP44 and SUGT1 serving as the predictive variables (**Figure 9A**). Calibration curve results demonstrated a strong alignment between the model’s actual and ideal diagnostic rates, confirming its favorable predictive performance; the model’s mean absolute error (MAE) and mean squared error (MSE) were 0.045 and 0.00312, respectively (**Figure 9B**). Furthermore, the nomogram model achieved internal and external validation AUC of 0.981 (95% confidence interval [CI]: 0.943–1) and 0.840 (95% CI: 0.517–1), respectively (**Figures 9C, D**). Collectively, these data verify that the prediction model exhibits acceptable accuracy and discriminative power, and the three key genes exhibit substantial clinical value for RPL diagnosis.

### 3.10 Construction of miRNA-mRNA regulatory networks for hub genes

**Figure 10:**
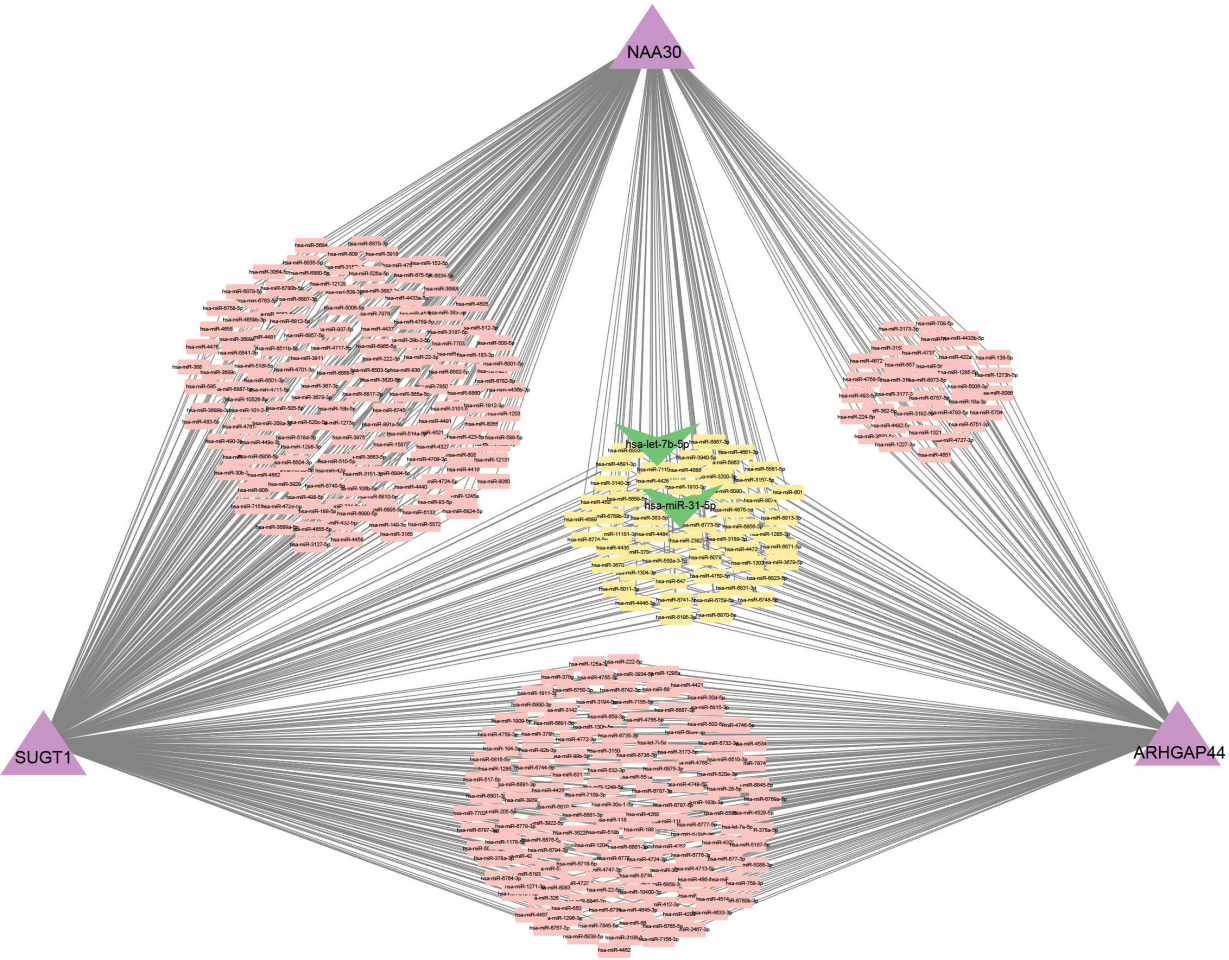
Construction of miRNA-mRNA regulatory networks for hub genes.

To further explore the underlying regulatory mechanisms of the three hub genes in RPL, we constructed an miRNA-mRNA regulatory network, which is illustrated in **Figure 10**. We retrieved miRNAs associated with the three hub genes from the miRWalk database, obtaining a total of 1, 375 candidate miRNAs. Among these, 52 miRNAs were commonly correlated with all three hub genes. Notably, hsa-let-7b-5p and miR-31-5p have been previously reported to be implicated in the regulation of trophoblast functions, leading us to hypothesize their potential involvement in the pathogenesis of RPL.

### 3.11 Validation of hub genes using human tissue samples

**Figure 11:**
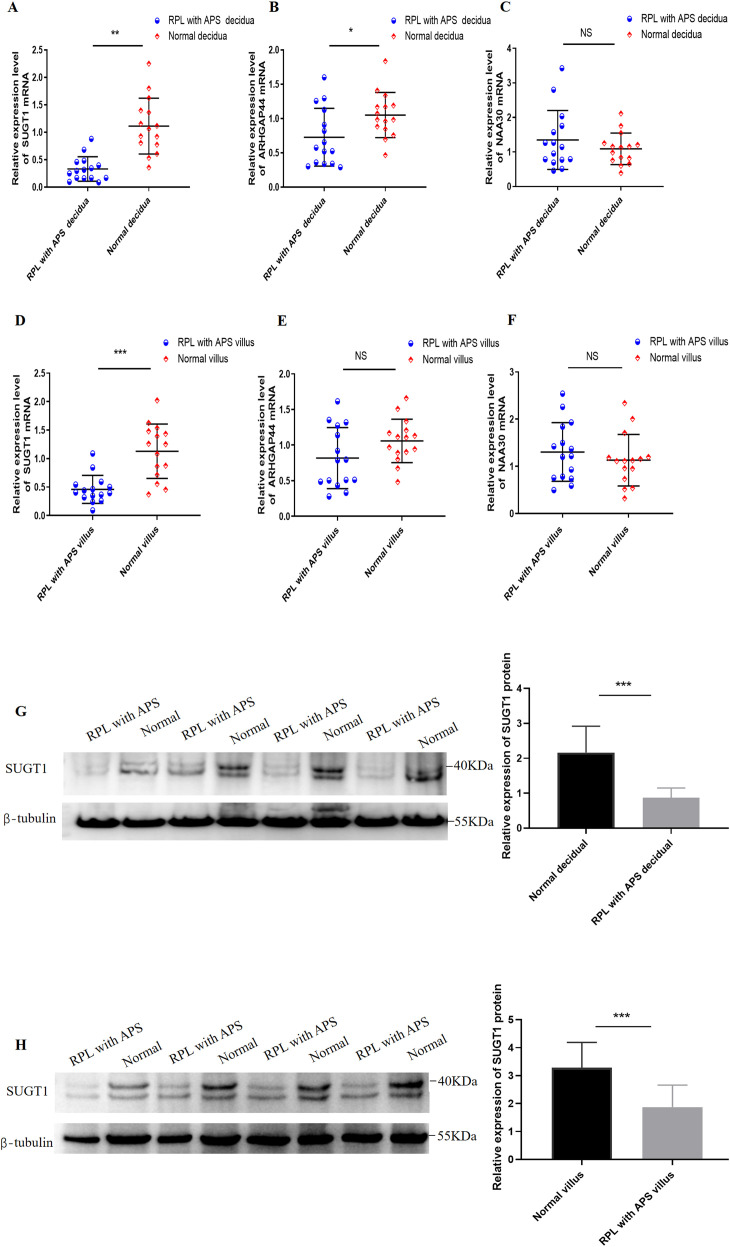
Low expression of SUGT1 in the decidual tissues of RPL with APS patients. **(A–C)** The expression levels of SUGT1, ARHGAP44, and NAA30 mRNA in decidual tissues were analyzed by qRT-PCR. **(D–F)** The expression levels of SUGT1, ARHGAP44, and NAA30 mRNA in villous tissues were analyzed by qRT-PCR. **(G)** The expression levels of SUGT1 protein in decidual tissues were analyzed by Western blot. **(H)** The expression levels of SUGT1 protein in villous tissues were analyzed by Western blot. (*P-value<0.05, **P-value<0.01, ***P-value<0.001).

To validate the expression patterns of NAA30, ARHGAP44, and SUGT1 in decidual tissues from patients with RPL, qRT-PCR was performed in the present study. As illustrated in **Figures 11A-C**, qRT-PCR results revealed that ARHGAP44 and SUGT1 exhibited a downward trend in mRNA expression in RPL tissues relative to normal decidual tissues. This observation was largely consistent with the findings derived from our prior data analysis, with SUGT1 demonstrating the most prominent reduction in RPL samples. However, the downregulation of NAA30 mRNA expression in decidual tissues of RPL patients failed to reach statistical significance. A potential contributing factor to this outcome may be the limited sample size employed in the current study. We further investigated the expression of these three genes in villous tissues. The results showed that only the expression profile of SUGT1 was consistent with that in decidual tissues, which also exhibited a significant reduction. By contrast, the downregulation of ARHGAP44 and NAA30 mRNA expression in RPL villous tissues did not reach statistical significance(**Figures 11D–F**). Given that SUGT1 exhibited prominent downregulation in both decidual and villous tissues of RPL patients, we further detected and compared its protein expression levels in decidual and villous samples from RPL patients and healthy controls. Consistent with the aforementioned findings, the protein level of SUGT1 was also significantly downregulated in both decidual and villous tissues of RPL patients.(**Figures 11G, H**).

### 3.12 The role of SUGT1 in the proliferation, migration, and invasion of trophoblast cells

**Figure 12:**
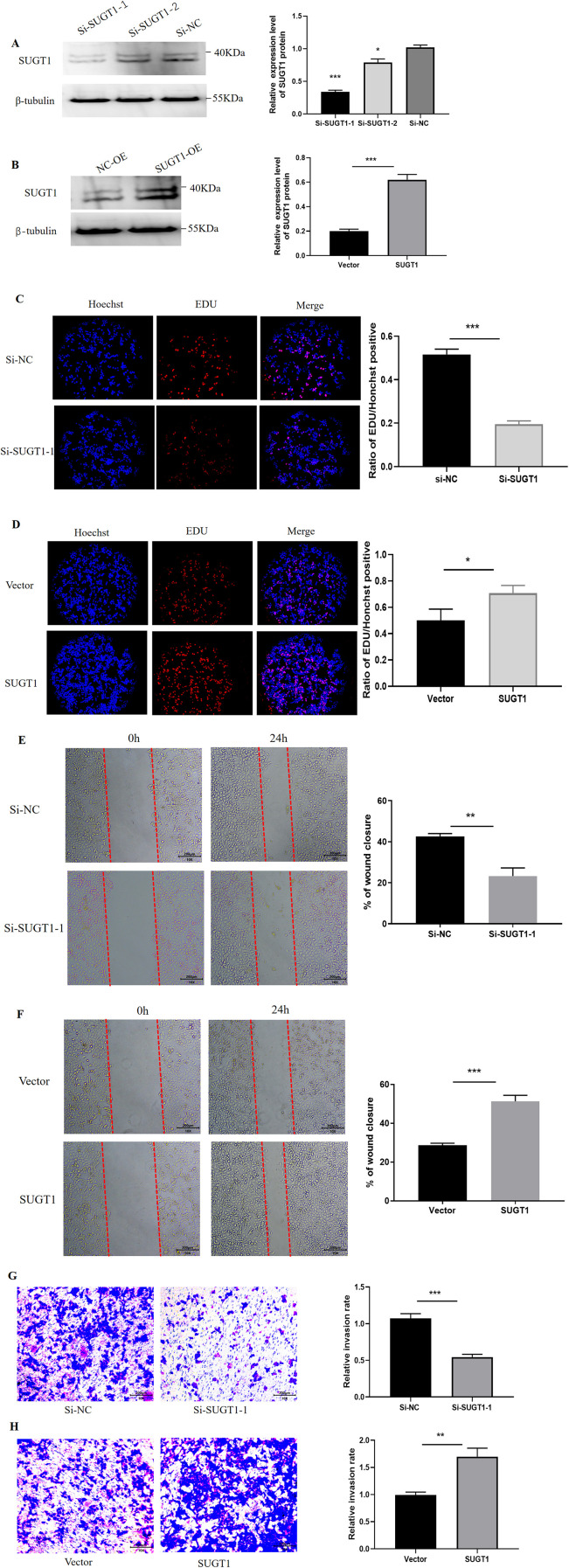
The role of SUGT1 in the proliferation, migration, and invasion of trophoblast cells. **(A)** After SUGT1 siRNAs (Si-SUGT1-1, Si-SUGT1-2) were transfected individually, relative expression of SUGT1 in trophoblast cells was detected by Western blot assays. The results showed that Si-SUGT1–1 is more effective, so we selected Si-SUGT1–1 for all subsequent experiments. **(B)** Overexpression of SUGT1 was validated by Western blot. **(A–D)** EdU assay was performed to assess the proliferation of cell. Red (EdU) HTR8/SVeno cells indicated proliferating the cell nucleus and blue (Hoechst 33342) represented the cell nucleus. **(E, F)** Cell migration was determined by *in vitro* wound-healing assay. **(G, H)** Transwell assay was conducted to measure the cell invasion ability. (*P-value<0.05, **P-value<0.01, ***P-value<0.001).

We further explored the biological role of SUGT1 in trophoblast cells. To this end, we manipulated SUGT1 expression in HTR-8/SVneo cells through siRNA-mediated knockdown or plasmid-based overexpression. (**Figures 12A, B**). Functional assays revealed that while SUGT1 silencing significantly inhibited trophoblast proliferation, migration, and invasion, SUGT1 overexpression exerted the opposite effect, enhancing these cellular capabilities (**Figures 12C–H**).

## 4 Discussion

This study took the GEO database as the core data source, systematically searched and extracted the associated datasets of APS and RPL. Through cross-validation by integrating multiple bioinformatics analysis methods, 10 genes with common differential expression in both diseases were finally screened out. This result provides a core target pool for further exploring the common pathological molecular basis of APS and RPL, and also confirms that there is a close internal connection between the two diseases at the gene expression level, which echoes the epidemiological characteristic that APS patients have a high incidence of RPL in clinical practice at the molecular level. To identify hub genes with diagnostic value, we applied three machine learning algorithms (LASSO, GMM, RF) combined with nomogram validation, and identified NAA30, ARHGAP44, and SUGT1 as key downregulated hub genes connecting APS and RPL.

Classical theory links APS-associated RPL to antiphospholipid antibody-induced thrombosis, but accumulating evidence emphasizes immune cell (especially T/B cell) dysregulation as a key pathogenesis (12, 17). This suggests that NAA30, ARHGAP44, and SUGT1 may play a “bridge” role in the pathological process of APS-induced RPL by regulating the related immune pathways. Our study confirmed these hub genes are involved in arginine/proline metabolism, alpha-linolenic acid metabolism, and linoleic acid metabolism across disease cohorts. Their co-enrichment in inflammation-related pathways is closely associated with immune regulatory networks, which directly explains the molecular mechanism underlying the link between APS and RPL.

CIBERSORT-based immune infiltration analysis suggested potential differences in immune cell proportions in APS and RPL. Immune cell changes disrupt maternal-fetal homeostasis, with quantitative or functional abnormalities linking the two conditions. Our study showed that APS patients had higher frequencies of naive CD4^+^ T cells but lower frequencies of resting memory CD4^+^ T cells. RPL patients had fewer activated dendritic cells, possibly impairing antigen presentation and immune tolerance. Notably, neutrophils in APS patients correlated negatively with other immune cells, indicating that neutrophils play a key pathogenic role in APS-related RPL, consistent with the reported mechanism of NETs-mediated placental injury (18, 19).

CTD analysis confirmed that these hub genes are closely associated with placental dysfunction and adverse pregnancy outcomes. Given the critical role of trophoblast function in placental formation, its proper regulation is pivotal for the development, maturation, and functional integrity of the placenta. APS is widely acknowledged as a common etiological factor for adverse pregnancy outcomes, as it impairs trophoblast function. Such functional impairment disrupts both the structural integrity and physiological functionality of the placenta, thereby contributing to RPL, preterm birth, and other pregnancy-related complications (20–23). Our findings therefore support that the three hub genes participate in APS-related RPL by regulating placental and trophoblast function. Furthermore, miRNA-mRNA integration analysis predicted hsa-let-7b-5p and miR-31-5p as core regulatory molecules in RPL, which synergistically target three hub genes. Existing studies have confirmed that hsa-let-7b regulates trophoblast invasion and migration (24) and maintains functional homeostasis via the ERK1/2 pathway (25). Similarly, miR-31-5p is also known to regulate trophoblast biological functions during placental development (26, 27), confirming its critical role in placental development. Thus, we speculate that aberrant hsa-let-7b-5p/miR-31-5p may exacerbate placental dysfunction by coordinately regulating the hub genes, leading to RPL.

Based on our miRNA-mRNA regulatory network analysis, hsa-let-7b-5p and miR-31-5p were identified as potential upstream regulators that may directly target SUGT1. Previous studies have confirmed that both miRNAs play critical roles in regulating trophoblast proliferation, migration, and invasion (24, 26), which are closely associated with the pathogenesis of recurrent pregnancy loss. Therefore, we speculate that these two miRNAs may contribute to the downregulation of SUGT1 in APS-related RPL by directly inhibiting its expression.

We further validated the three hub genes using clinical decidual and villous tissues from APS-related RPL patients.ys. Results showed that among the three hub genes, SUGT1 was significantly downregulated in both decidual and villous tissues of these patients. On this basis, SUGT1 was selected as the target for subsequent in-depth investigation. Consistent with the RT-qPCR data, Western blot analysis further confirmed that SUGT1 protein expression was significantly decreased in both decidual and villous tissues, thereby validating the reliability of our initial bioinformatics-based discoveries. *In vitro* experiments were conducted using the human trophoblast cell line HTR8/SVeno. Functional assays further demonstrated that SUGT1 plays a pivotal role in regulating the biological behaviors of HTR8/SVeno cells, as it significantly inhibits their proliferation, migration, and invasion. Given that abnormal proliferation, migration, and invasion of trophoblast cells are core pathological features of RPL (28), these findings suggest that SUGT1 may act as a key mediator in the pathogenesis of RPL-related trophoblast abnormalities.

This study has several limitations. First, the small clinical sample size may limit the generalizability of our findings, which requires further validation in larger cohorts. Second, machine learning models carry potential overfitting risks that should be considered during result interpretation. Third, although the expression and function of hub genes were verified by qPCR, Western blotting, and *in vitro* experiments, additional *in vivo* studies are needed to clarify their exact regulatory mechanisms. Ultimately, the detailed regulatory network of SUGT1 in HTR-8/SVneo cell proliferation, migration, and invasion warrants further exploration. Despite these limitations, our study provides novel hub genes and mechanistic insights into APS−related RPL, which may facilitate the development of new diagnostic biomarkers and therapeutic targets.

## 5 Conclusions

This study preliminarily uncovers the candidate hub genes in RPL with APS. These genes were validated through multiple experimental methods, including RT-qPCR and Western blotting, with additional support from bioinformatic analyses. Specifically, NAA30, ARHGAP44 and SUGT1 are involved in the pathophysiological processes of RPL with APS, with their roles mediated through mechanisms such as insufficient metabolic support, enhanced immune aggression and impaired cellular function. These results may provide a basis for developing early diagnostic approaches, prognostic indicators and therapeutic targets for RPL with APS.

## Data Availability

The original contributions presented in the study are included in the article/**Supplementary Material**. Further inquiries can be directed to the corresponding author.

## Supplementary material

Supplementary Figure 1 GSEA of individual diagnostic genes. **(A, B)** GSEA of ARHGAP44 in the APS and RPL cohorts. **(C, D)** GSEA of NAA30 in the APS and RPL cohorts. **(E, F)** GSEA of SUGT1 in the APS and RPL cohorts.

Supplementary Figure 2 Correlation analyses between hub genes and immune cell infiltration. **(A, B)** Correlation scatter plots of ARHGAP44 and immune cells in APS **(A)** and RPL **(B)**. **(C, D)** Correlation scatter plots of NAA30 and immune cells in APS **(C)** and RPL **(D)**. **(E, F)** Correlation scatter plots of SUGT1 and immune cells in APS **(E)** and RPL **(F)**. (Data with P < 0.05 were highlighted).

Supplementary Table 1 Baseline characteristics of study subjects.

Supplementary Table 2 Primers used for qRT-PCR.

Supplementary Table 3 The list of genes in Co-Expression Modules in RPL.

Supplementary Table 4 The gene expression levels of 42 key genes in RPL.
