# Supplementary figures and images for: Integrative analysis of hub genes for recurrent pregnancy loss with antiphospholipid syndrome: integrated bioinformatics analysis, machine learning and experimental validation
Source: Front Immunol. 2026 Jun 4;17:1783244. doi: 10.3389/fimmu.2026.1783244 (PMC13275653; doi:10.3389/fimmu.2026.1783244)

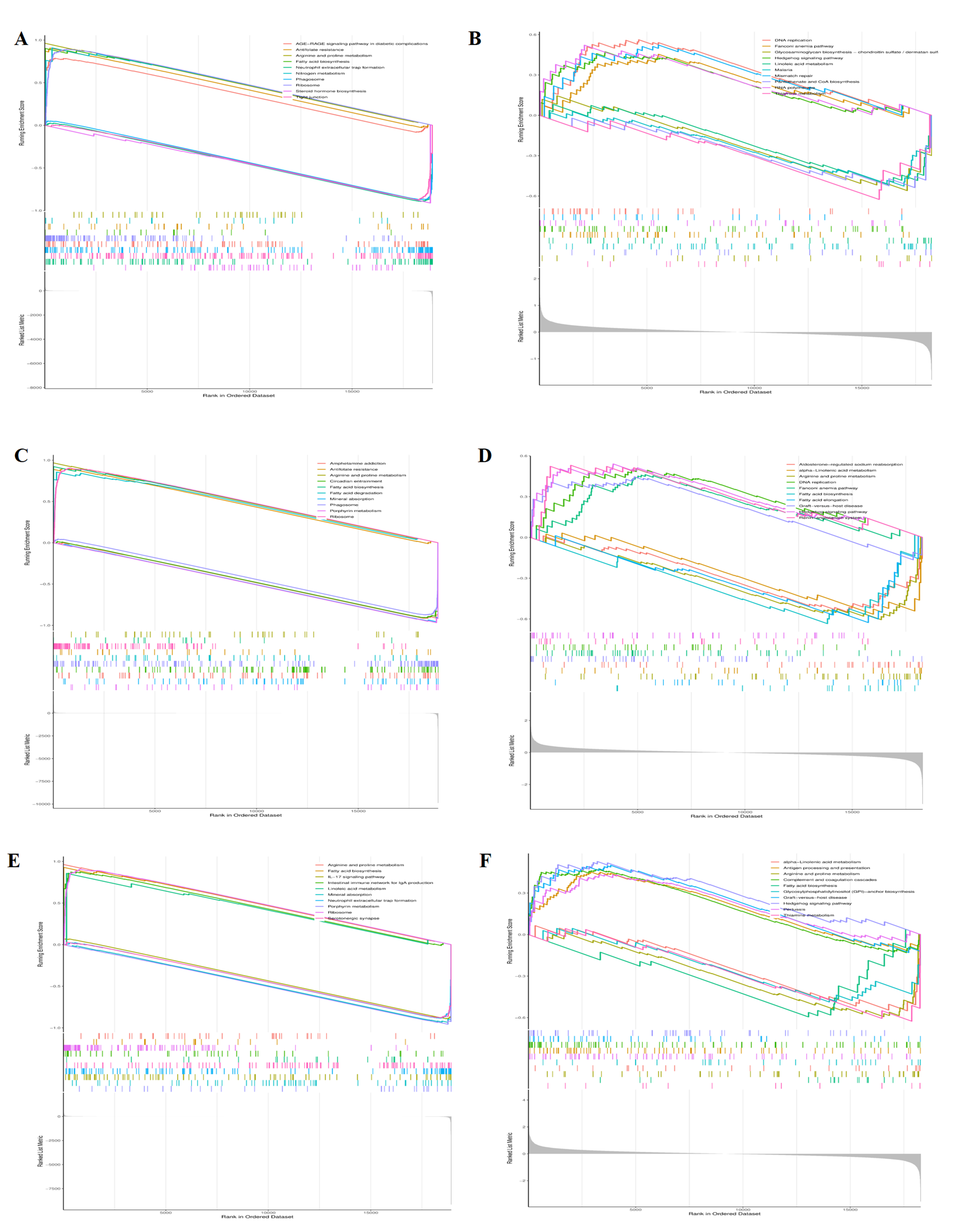

Supplement: Supplementary Figure 1 — GSEA of individual diagnostic genes. (A, B) GSEA of ARHGAP44 in the APS and RPL cohorts. (C, D) GSEA of NAA30 in the APS and RPL cohorts. (E, F) GSEA of SUGT1 in the APS and RPL cohorts. [file Image1.tif]

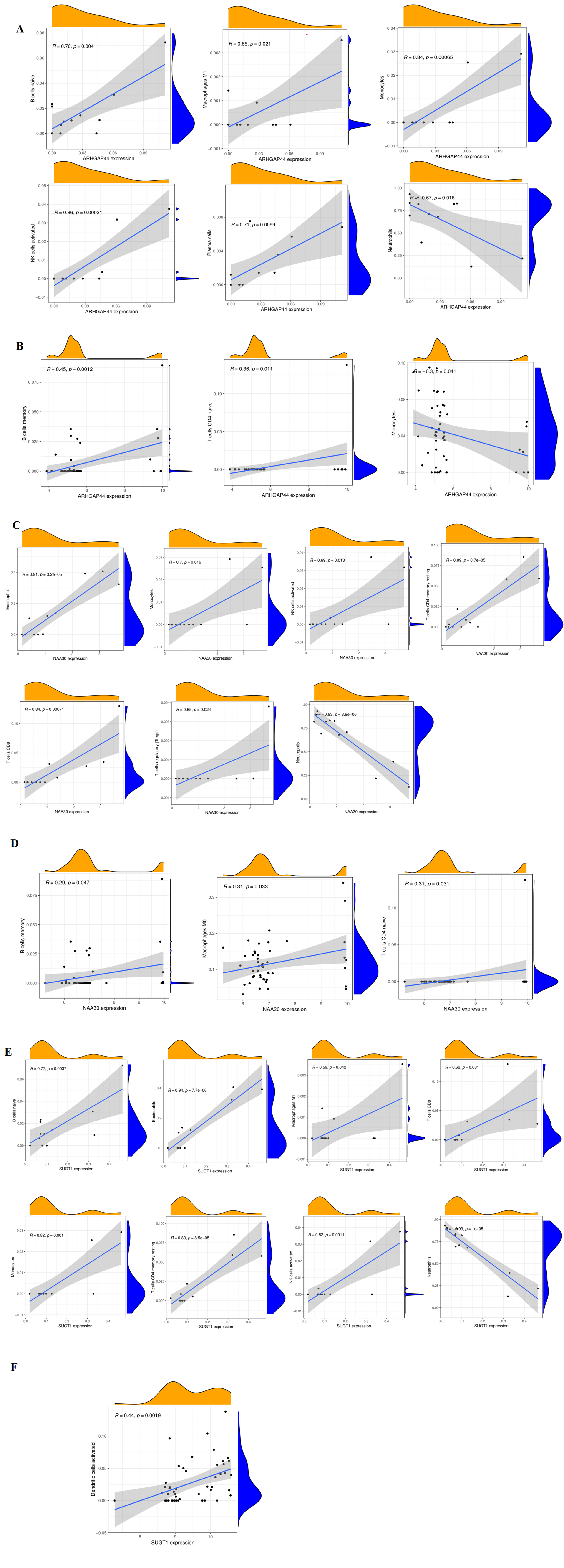

Supplement: Supplementary Figure 2 — Correlation analyses between hub genes and immune cell infiltration. (A, B) Correlation scatter plots of ARHGAP44 and immune cells in APS (A) and RPL (B). (C, D) Correlation scatter plots of NAA30 and immune cells in APS (C) and RPL (D). (E, F) Correlation scatter plots of SUGT1 and immune cells in APS (E) and RPL (F). (Data with P < 0.05 were highlighted). [file Image2.tif]
