# Supplementary material for: Integrative analysis of hub genes for recurrent pregnancy loss with antiphospholipid syndrome: integrated bioinformatics analysis, machine learning and experimental validation
Source: Front Immunol. 2026 Jun 4;17:1783244. doi: 10.3389/fimmu.2026.1783244 (PMC13275653; doi:10.3389/fimmu.2026.1783244)
Supplement: Supplementary Table 1 — Baseline characteristics of study subjects. [file Table1.doc]

**Supplementary Table 1. Baseline characteristics of study subjects**

|  | APS+RPL  (n=15) | Normal  (n=15） | P value |
| --- | --- | --- | --- |
| Age (year) | 34.26±3.86 | 32.93±3.75 | 0.345 |
| gestation age | 8 (7-9) | 7 (7-8) | 0.233 |
| body mass index（BMI）(kg/m2) | 20.56±1.81 | 20.72±1.05 | 0.776 |
| APL positive, n (%) |  |  |  |
| Lupus anticoagulant (LA) | 8 (53.33) | 0 | ＜0.01 |
| Anti-cardiolipin (aCL) | 9 (60.00) | 0 | ＜0.01 |
| Anti-β2GPI | 7 (46.67) | 0 | ＜0.01 |
