# Supplementary material for: Integrative analysis of hub genes for recurrent pregnancy loss with antiphospholipid syndrome: integrated bioinformatics analysis, machine learning and experimental validation
Source: Front Immunol. 2026 Jun 4;17:1783244. doi: 10.3389/fimmu.2026.1783244 (PMC13275653; doi:10.3389/fimmu.2026.1783244)
Supplement: Supplementary Table 2 — Primers used for qRT-PCR. [file Table2.doc]

**Supplementary Table 2.** Primers used for qRT-PCR

| **Name** | **Forward** | **Reverse** |
| --- | --- | --- |
| NAA30 | CCCTCAAGAGCAAGGTCCTG | GGCTCCTTTTGTTGCAGTCG |
| ARHGAP44 | ATTTGCAGGGACCAGCTCTC | CTTCTCTACCCAGGCCTCCT |
| SUGT1 | TGTGTTGCTGTTGCTGATGC | ACACGATGAAACCCCGTCTC |
| GAPDH | CAGGAGGCATTGCTGATGAT | GAAGGCTGGGGCTCATTT |
