# Supplementary material for: Integrative analysis of hub genes for recurrent pregnancy loss with antiphospholipid syndrome: integrated bioinformatics analysis, machine learning and experimental validation
Source: Front Immunol. 2026 Jun 4;17:1783244. doi: 10.3389/fimmu.2026.1783244 (PMC13275653; doi:10.3389/fimmu.2026.1783244)
Supplement: Supplementary Table 3 — The list of genes in Co-Expression Modules in RPL. [file Table3.doc]

**Supplementary Table 3** The list of genes in Co-Expression Modules in RPL

| Module | Gene |
| --- | --- |
| grey60 module | OR4D11, LEO1, OR6K2, SNRPC, XLOC_001265, TAF5L, LOC643529, ARHGEF25, XLOC_008465, XLOC_l2_006025, XLOC_004197, LOC148709, XLOC_007460, IDH3A, LOC729815, XLOC_009110, C17orf105, ICMT, XLOC_008216, XLOC_014396, RABEPK, SNORA71C, XLOC_009659, ATP8B5P, RPS7P5, XLOC_l2_007449, SNORA71B, FKSG2, PSD4, XLOC_l2_011620, FLJ37201, PGAM4, SLC6A17, SUGT1, ATMIN, EHBP1, XLOC_013994, CDK11B, SNRPE, C18orf32, CKLF, FAM199X, XLOC_l2_003065, EIF2S2, PDK2, XLOC_l2_007829, ND4, GTSCR1, RNU2-2, XLOC_l2_015938, RBMXL1, OR10G4, OAS1, DDX6, C1orf140, MTRNR2L8, TPM3, HRASLS5, PSIP1, SUMO1P3, ANXA2, NAA30, FEZ2, ETF1, F2R, WBP11, CAPZA2, TCP1, ZNF90, KIAA0319L, GLI4, BOLA3, ZNF729, MRPS36, ZNF295, CASKIN1, LOC647979, SET |
| brown module | MLKL, IFITM2, TBC1D16, KIAA0922, HDHD3, GLIS2, LINC00458, C1orf162, GYS1, MCAM, MAN2A1, EGLN3, SOX5, ABO, ALAD, ENHO, PCDHB16, IFITM4P, LOC100506609, XLOC_002296, LOC100507673, RASSF7, ITGB5, COL16A1, ANKRA2, LOC145820, HLA-DPA1, SLC2A6, PSD, NLRC4, XLOC_l2_009968, C2orf29, KLRB1, ARHGAP20, ARHGEF35, PPARG, CNN2, XLOC_004598, SLC4A3, Q5A5F0, CTHRC1, FLJ46120, C1QA, GRM7, KRT18, SYT7, XLOC_009363, DGKB, CMTM6, ZNF618, TREX1, NAA60, XLOC_005621, XLOC_003775, LOC100128593, SLC23A1, RSPO1, PRKCB, WTIP, LENG9, AMOTL2, ATP6V1C2, XLOC_003200, IFIT3, AMDHD1, DUSP14, MIR31HG, ATP6V1G1, FSTL1, PGM2L1, CTAGE15P, SMARCD3, CARD16, ACOX3, GIMAP7, TLE3, TMEM47, SPINK6, FCGR2C, ALDH1A2, P4HTM, XLOC_l2_004870, LOC645591, TMEM71, SLC22A18, RHOD, ANXA10, PDE3B, ABCA2, ARRDC1, ADAMTS3, STRA6, MS4A7, IGSF6, CPEB2, CIB4, MDFI, OSBPL2, HOTAIRM1, HCP5, NCF2, TNFRSF13C, XLOC_012139, LOC729799, THAP4, CSRNP1, WNT2, XLOC_l2_002110, XLOC_l2_003757, ENPP2, CTAGE11P, C1QB, AGPS, ZNF776, PLCB1, GOLPH3, EPHB2, LOC100507376, C10orf81, PLD1, IRF6, LOC100288092, XLOC_l2_015202, CREG1, CDC14B, HLA-DRA, C20orf141, XLOC_013461, BAG2, LOC100240735, TNFRSF11A, C6orf183, XLOC_000387, FRZB, SAMD11, KRT18P55, TSN, LILRB2, XLOC_l2_013149, TAF7L, HLA-DRB3, WWC1, RAB20, TGFB3, HLA-DRB4, PTEN, TLR2, SH3GLB2, GPRIN1, TMPRSS2, XLOC_009764, MGC34796, XLOC_004956, PI16, CLSTN2, ATP6V1G2, LOC100507025, ARL4C, SERTM1, FRK, FAM26F, PRDM6, TSPAN33, TLR7, AK7, USP9X, HPDL, COL24A1, XLOC_013931, BNC2, C1orf151-NBL1, PTPRO, GBP5, CNDP2, RGS18, VPS37B, PRSS23, SLIT2, KCNK6, PDE8B, MYBPC2, CMYA5, NRXN3, IL4I1, ATP8A1, WASH3P, FGF1, C1QTNF5, SOX4, PLIN4, C9orf152, CCND2, ALDH6A1, XLOC_010997, RBP1, C2orf88, EDIL3, CPNE4, LOC100130547, VNN2, TMEM101, SGK223, RHOB, TMEM132E, GBP3, ARL4D, XLOC_009191, ARL6IP1, NPTN, MYRIP, CDKN1B, LUM, XLOC_007867, IL28RA, BTNL9, PLAUR, TEK, SGIP1, PDLIM3, C7orf41, LOC100507186, HOOK1, MNDA, RASSF10, FN1, RND3, IL17D, SHROOM3, GNG2, RNF182, ADAMTS6, STXBP2, SLC9A3R2, AADAC, DAPK2, LOC283516, PION, RBM3, SEZ6L, TMEM141, PIR, ADK, XLOC_000462, SLC25A29, BDH1, L3MBTL4, IL8, PHF8, DNMBP-AS1, TNFRSF12A, ACSM1, ASPN, IGSF10, HNRNPH3, ZNF600, LYN, WNK4, PKD1L2, CTH, ARHGAP22, ARSF, NQO1, DLG5, XLOC_012610, GNMT, CSF3R, FAM195A, ARHGEF5, IL1R2, MEI1, CRYGN, XLOC_010856, NEURL, XLOC_005217, GREM1, C6orf226, SAT1, FCGR3A, SLC43A3, SNCA, MX1, LOC100499183, NAT8, XLOC_011736, XLOC_007214, ELMO2, ITGBL1, KIAA1671, RAB15, CST6, ERLEC1, NOD2, ANKRD35, BMPER, HMOX1, SETDB1, BCL2L13, SMTNL2, RAMP2, WNT6, DCXR, PLD5, LOC100499467, RNF125, DKK2, MRVI1, CAMK2D, LRRC2, CYB5A, ZSWIM4, BST2, NFE2, PAPSS1, PRKAG2, XLOC_l2_005076, FAM78B, XLOC_005211, PDPN, KIAA0226L, PRDM1, LOC100506295, KCNJ12, MOGAT1, BMP7, ST3GAL5, SLC26A3, ACSM3, ISM1, REN, CFB, HLA-DRB5, TPPP, TXNDC16, LOC100131726, PIKFYVE, CKMT2, XLOC_001243, WDR3, GJA1, RSPO3, PARL, XLOC_l2_013125, KCNJ18, INHBA, PSORS1C3, XLOC_009249, PLG, CCBL1, TDRD9, METTL23, LCN2, TSC2, CA4, OCA2, FSTL3, CST2, RANBP17, CTSC, ELP3, AIM1L, PRKY, STX19, PRKX, GRHL2, IL1RN, MUC16, LOC729995, ATP2B2, PTGS2, XLOC_010599, MFSD6, KIAA2013, HOMER2, KRT8P12, XLOC_003202, SIDT1, CST1, MBD6, OVOL1, PPM1H, SNTG2, CCDC80, COL12A1, LRRC1, FLRT3, CREB3L1, CKB, FAM150B, THBS1, HAPLN1, ROR2, DENND2C, ORM1, FAM5B, NKAIN1, MOV10, EWSR1, LRP4, HPR, GMPR, ERN2, XLOC_001257, LOC100506428, COBL, FAM107A, XLOC_002867, XPO6, PLA2G4F, AZGP1, XLOC_004685, TFCP2L1, JHDM1D, SULT1E1, CD86, CABYR, ALDH3B2, HP, SRSF7, CDS1, CNN1, FOLR1, XLOC_009382, TFPI2, XLOC_001699, SUMO1, KDM4C, SLC25A48, GRHL1, BIRC3, PNMT, PTK2, XLOC_008559, AP3S1, PDE6A, COL4A6, PKDCC, ERBB4, C19orf77, NPR3, FAM155B, DISP1, C11orf9, CHST15, MLLT1, ABCA12, NLRP5, PDZK1, TNK2, XLOC_l2_014865, ADAMTS8, NOTCH2, CHST4, PLA2G4D, PEBP4, COL5A1, TM4SF4, P4HA2, PTPRJ, SCGB1D4, OPRK1, DUOXA1, HLA-DOB, LRRC26, DUOX1, SLAIN1, TSEN2, AK4, LOC100507008, MPZL2, CAPN6, LOC100505912, XDH |
