# Supplementary material for: Integrative analysis of hub genes for recurrent pregnancy loss with antiphospholipid syndrome: integrated bioinformatics analysis, machine learning and experimental validation
Source: Front Immunol. 2026 Jun 4;17:1783244. doi: 10.3389/fimmu.2026.1783244 (PMC13275653; doi:10.3389/fimmu.2026.1783244)
Supplement: Supplementary Table 4 — The gene expression levels of 42 key genes in RPL. [file Table4.doc]

**Supplementary Table 4** The gene expression levels of 42 key genes in RPL

| **Id** | **logFC** | **P Value** |
| --- | --- | --- |
| ZNF90 | 3.22747 | 5.40E-33 |
| HRASLS5 | 1.85385 | 6.67E-17 |
| CASKIN1 | 3.79053 | 3.73E-16 |
| SNRPE | 1.31707 | 4.59E-15 |
| TCP1 | 2.50359 | 1.39E-12 |
| TPM3 | 1.49286 | 1.47E-09 |
| SNORA71B | 1.01926 | 2.05E-09 |
| ZNF295 | 2.88283 | 6.56E-08 |
| RAMP2 | 1.23754 | 2.02E-06 |
| ZNF729 | 2.50824 | 3.40E-06 |
| GLI4 | 2.2447 | 7.19E-06 |
| FEZ2 | 1.60664 | 1.20E-05 |
| PAPSS1 | 1.13184 | 2.73E-05 |
| ELMO2 | 1.03395 | 5.97E-05 |
| F2R | 1.5008 | 8.80E-05 |
| DKK2 | 1.01496 | 0.000244093 |
| ZSWIM4 | 1.00995 | 0.000331796 |
| MOV10 | 1.39814 | 0.000349649 |
| EWSR1 | 1.3741 | 0.000459913 |
| METTL23 | 1.0876 | 0.000788255 |
| WDR3 | 1.0444 | 0.000895345 |
| KRT8P12 | 1.11419 | 0.001693913 |
| MPZL2 | 2.52186 | 0.001908572 |
| PNMT | 1.49158 | 0.002091049 |
| FAM155B | 1.6219 | 0.002881724 |
| SIDT1 | 1.0602 | 0.003315489 |
| FAM107A | 1.14888 | 0.004939129 |
| CAPN6 | 2.35158 | 0.00577919 |
| NPR3 | 1.47241 | 0.007165892 |
| NOTCH2 | 1.59814 | 0.007276624 |
| FOLR1 | 1.1497 | 0.012563226 |
| SULT1E1 | 1.03765 | 0.017576932 |
| SCGB1D4 | 1.52424 | 0.021784069 |
| ADAMTS8 | 1.34091 | 0.022480176 |
| PDE6A | 1.15997 | 0.023247713 |
| C19orf77 | 1.22515 | 0.024128675 |
| LOC100505912 | 1.94521 | 0.025895738 |
| TM4SF4 | 1.38908 | 0.035568831 |
| CHST4 | 1.29377 | 0.037512322 |
| HLA-DOB | 1.41352 | 0.039798411 |
| LRRC26 | 1.41878 | 0.042323251 |
| PLA2G4D | 1.25173 | 0.045537934 |
